# Microbial-Driven Butyrate Regulates Jejunal Homeostasis in Piglets During the Weaning Stage

**DOI:** 10.3389/fmicb.2018.03335

**Published:** 2019-01-18

**Authors:** Xi Zhong, Zhongwei Zhang, Shujin Wang, Lili Cao, Lin Zhou, Aomin Sun, Zhendong Zhong, Miranda Nabben

**Affiliations:** ^1^Intensive Care Unit, West China Hospital, Sichuan University, Chengdu, China; ^2^Department of Genetics and Cell Biology, Faculty of Health, Medicine and Life Sciences, Maastricht University, Maastricht, Netherlands; ^3^Medical School, Chengdu University, Chengdu, China; ^4^Shenzhen Premix Inve Nutrition, Co., Ltd., Shenzhen, China; ^5^Chengdu LiLai Biotechnology, Co. Ltd., Chengdu, China

**Keywords:** *Sus scrofa*, weaned, jejunum, microbiota, butyrate, inflammatory response, apoptosis, proliferation

## Abstract

Microbe-derived butyrate plays an important role in the gut health of young mammals during the weaning stage. A greater understanding of how butyrate regulates intestinal development is necessary for overcoming post-weaning diarrheal diseases. We aimed to investigate whether jejunal microbial metabolite butyrate modulates the apoptosis/proliferation balance and immune response in piglets during the post-weaning period of the first 3 weeks of life. On the one hand, during the first week post-weaning, the relative abundances of the dominant bacterial families *Erysipelotrichaceae* (*P* < 0.01) and *Lachnospiraceae* (*P* < 0.01) were increased, which induced decreases in both butyrate production (*P* < 0.05) and its receptor (G-protein coupled receptor 43) expression (*P* < 0.01). The resulting intestinal inflammation (inferred from increased TNF-α and IFN-γ expression) contributed to the onset of cell apoptosis and the inhibition of cell-proliferation along the crypt-villus axis, which were followed by impaired jejunal morphology (i.e., increased crypt-depth) (*P* < 0.05) and intestinal dysfunction (i.e., decreased creatine kinase, and lactate dehydrogenase) (*P* < 0.05). On the other hand, during the second week post-weaning, the relative abundances of *Lactobacillaceae* (*P* < 0.01) and *Ruminococcaceae* (*P* < 0.05) were increased. The increases were accompanied by increased butyrate production (*P* < 0.05) and its receptor expression (*P* < 0.01), leading to the inhibition of cell apoptosis and the stimulation of cell proliferation via decreased pro-inflammatory cytokines and thereby the improvement of intestinal development and function. Herein, this study demonstrates that microbial-driven butyrate might be a key modulator in the maintenance of intestinal homeostasis after weaning. The findings suggest that strategies to promote butyrate production can maintain the apoptosis/proliferation balance via minimizing intestinal inflammation, and thereby improving post-weaning jejunal adaptation toward gut health.

## Introduction

The moment of introduction of solid food instead of breast milk is highly important for pigs due to the high risk of diarrheal diseases (Lallès et al., 2007b). This so-called weanling diarrhea is accompanied by gastrointestinal disturbances, such as disturbed absorptive-secretory electrolyte balance, transiently increased mucosal permeability and altered local inflammatory cytokine patterns, which can be attributed to large shifts in intestinal microbial composition (Pie et al., 2004; Lallès et al., 2007a). Although improvements in nutrition, health and management have been considered to minimize the adverse effects induced by weaning stress, a greater understanding of the biological impacts of stress is necessary for overcoming diarrheal diseases (Campbell et al., 2013).

Butyrate is one of the major short-chain fatty acids (SCFAs) and is produced by microbial fermentation in the gastrointestinal tract (GIT) (Wang et al., 2011). In addition to being used as a substrate for energy production, butyrate promotes cell proliferation and inhibits cell apoptosis, leading to the functional maturation of intestinal epithelial cells (Hamer et al., 2008). Furthermore, butyrate can mediate the effects of intestinal microbiota on the host’s appetite, metabolism, adiposity, and immunity (Meijer et al., 2010; Wang et al., 2011). Intriguingly, intestinal epithelial cells undergo functional and morphological differentiation during migration along the crypt-villus axis (Mariadason et al., 2005; Xiong et al., 2015). As a result, the turnover (i.e., proliferation, differentiation, and apoptosis) of intestinal epithelial cells is essential for mucosal development, maintenance, and recovery from tissue damage during the suckling-weaning transition period (Yang et al., 2013). However, the mechanisms underlying the signaling effects of butyrate on the apoptosis and proliferation of intestinal epithelial cells along the crypt-villus axis have not yet been elucidated.

To date, most attention has been focused on fecal and colonic microbiota, but the microbiota in the jejunum has been shown to be essential for the host’s physiology, encompassing metabolic, immune and endocrine functions (Magne et al., 2006; Palmer et al., 2007; El Aidy et al., 2013, 2015; Matamoros et al., 2013). Despite its low microbial densities, the jejunum responds strongly to the presence of microbiota (El Aidy et al., 2015). In a time-course study of metabolic responses of GIT mucosa to conventionalization, a rapid and dynamic response was observed in the jejunum that was not observed in other parts of the GIT, including the ileum and colon, supporting a unique role of the jejunum in response to the luminal microbiota-diet interplay (El Aidy et al., 2013). Therefore, insight into the role of the jejunum in microbiota-host interactions is necessary to explore strategies for protecting vulnerable individuals against post-weaning diarrhea and infection in piglets (Wang et al., 2015a,b). The objective of this study is to investigate whether the microbial metabolite butyrate regulates mucosal adaption in the jejunum of piglets after weaning.

## Materials and Methods

### Animal Experiments

The animal care and procedures used in this study were performed according to the guidelines of the China Animal Protection Association and were approved by the Shenzhen Animal Care Committee, Shenzhen, China.

### Piglets and Feeding

In total, 40 conventionally raised Landrace-Duroc piglets were randomly assigned to five pens of eight piglets each. The piglets had been weaned at a 300-sow batch-farrowing facility (Shenzhen Premix Inve Nutrition, Co., Ltd., Shenzhen, China) at post-natal (PN) day 28. Each piglet had a unique identifier assigned at weaning. The piglets were randomly selected, had an average initial body weight (BW) of 6.38 ± 0.01 kg, and were healthy without any clinical signs of disease. The piglets were fed a standard diet (Supplementary Table S1) formulated as a powder and without any in-feed antibiotics based on the guidelines of National Research Council (Cunha, 2012). All piglets had *ad libitum* access to feed and water, and the given and remaining feed was daily weighed at 0800 h. Average daily feed intake (ADFI) and BW were recorded daily and weekly, respectively, to calculate the feed-to-gain ratio (F/G) and average daily gain (ADG).

Individual daily clinical observations encompassing alertness and diarrhea scores were recorded daily beginning on PN day 28, i.e., post-weaning day 0. The standard alertness score ranged from 1 to 3 (1 = normal; 2 = slightly depressed; 3 = severely depressed). The daily individual diarrhea score ranged from 1 to 5 (1 = normal feces; 2 = moist feces; 3 = mild diarrhea; 4 = severe diarrhea; 5 = watery diarrhea) and was assessed by two independent evaluators. The frequency of diarrhea was recorded as a daily score of 3 or greater.

### Sample Collection and Processing

At the start of the post-weaning period (day 0) and at days 7, 14 and 21, five piglets at each time point were selected to sacrifice using an intravenous injection of sodium pentobarbital (approximately 150 mg/kg BW), and the jejunum of each was collected. Firstly, approximately 5 g of digesta from mid-jejunum were snap-frozen in liquid nitrogen and subsequently stored at -80°C for full length 16S rRNA and SCFAs analysis. Secondly, after slitting the jejunum lengthwise and gentle rinsing with ice-cold 1× PBS, the jejunal mucosa was scraped with a glass slide and rapidly placed in liquid nitrogen for measuring digestive enzymes, pro-inflammatory cytokines, signaling markers of cell apoptosis and cell proliferation, and SCFA receptors. Finally, an approximately 3.00-cm jejunal segment was rinsed with ice-cold 1× PBS and then fixed in 10% neutral formalin for measuring jejunal morphology and the immunohistochemistry of proliferating cell nuclear antigen (PCNA).

### Sequence Processing and Analysis of Jejunal Microbiota

#### DNA Extraction

In this study, total DNA was extracted from the jejunal contents with the E.Z.N.A.^®^ Stool DNA Kit (Omega Bio-tek, Inc., Norcross, GA, United States) in accordance with the manufacturer’s instructions. Quality of total DNA was verified using electrophoresis analysis.

#### Amplification and High-Throughput Sequencing of Jejunal Bacterial 16S rRNA

Amplification of full-length (V1–V9) 16S rRNA gene sequences was performed on a PacBio^®^ RS II platform (Majorbio Bio-Pharm Technology, Co., Ltd., Shanghai, China) (Yarza et al., 2014). Briefly, the full-length 16S rRNA genes were amplified by PCR using the universal bacterial primer set 27F (5′-AGAGTTTGATCCTGGCTCAG-3′) and 1492R (5′-GGTTACCTTGTTACGACTT-3′). The PCR reaction system was purchased from Beijing TransGen Biotech, Co., Ltd. (China), and PCR was carried out in a 50-μL reaction volume containing 25 ng DNA template, 0.40 mM (each) primer, 2.50 U Pfu polymerase and 0.25 mM dNTPs. The PCR conditions were as follows: initial denaturation at 94°C (4 min); 25 cycles of denaturation at 94°C (30 s), annealing at 55°C (30 s), and extension at 72°C (30 s); and a final extension at 72°C (10 min). To avoid bias, we conducted three independent PCRs for each individual sample in this study. After separation on an agarose gel (2% in TBE buffer), the PCR products were further purified with an AxyPrep DNA gel extraction kit (Axygen, Co., Hangzhou, China) for subsequent sequencing. Prior to sequencing, the purified PCR products were quantified using the QuantiFluor-ST fluorescence quantitative system (Promega, San Luis Obispo, CA, United States).

#### Bioinformatics

Raw Fastq files were demultiplexed and quality filtered using QIIME (version 1.17) in accordance with the following criteria (Zhu et al., 2015): (1) the 1494-bp reads were truncated at any site receiving an average quality score less than 20 over a 10-bp sliding window, (2) specific barcodes were exactly matched, (3) truncated reads of less than 50 bp were removed, (4) reads containing ambiguous characters were removed, (5) allowed mismatching with primers was at most 1 bp, (6) reads that could not be assembled were discarded, and (7) only sequences that overlapped by more than 10 bp were further assembled.

Operational taxonomic units (OTUs) were clustered with 97% similarity cutoff using UPARSE (version 7.1^1^), and chimeric sequences were identified and removed using UCHIME. RDP classifier was used for taxonomical assignments of each sequence at 70% confidence level using 16S rRNA sequences from Silva release 119^2^. Alpha diversities and rarefaction curves were assessed using Mothur (version v.1.30.1^3^). Community richness was assessed by Chao^4^ and ACE^5^ estimators, and community diversity was analyzed by the Simpson index^6^ and Shannon index^7^. Good’s coverage analysis was also evaluated in this study.

Bray–Curtis similarity clustering analysis was performed with the R programming language (version R 3.2.0). Briefly, non-metric multidimensional scaling (NMDS) was performed to indicate the relative associations among samples tested via orienting datasets in multidimensional space based on their dissimilarity or similarity at different growth stages after weaning (ggplot2 package). Venn diagrams were created with help of the VennDiagram package. A heatmap was generated from the differentially abundant taxa (x > 0.1% of the community), and a dual hierarchical clustering dendrogram was created using Vegan package. Furthermore, the linear discriminant analysis (LDA) effect size (LEfSe) algorithm was applied to identify core OTUs, and cladograms were analyzed with the LEfSe method^8^.

### Analysis of SCFA Concentrations

Approximately 300 mg jejunal samples were acidified to pH 2.2 using 25% metaphosphoric acid solution, and centrifuged at 4,800 × *g* for 20 min at 4°C and the internal standard (100 mM quinic acid) was added. After mixing by vortex for 30 s and standing at 20°C for 10 min, the centrifugation was repeated (4,800 *g*, 4°C for 20 min) and the supernatants were analyzed for SCFAs with an Agilent 1260 high-performance liquid chromatography system (Agilent Technologies, Inc., United States). All samples and SCFA standards (i.e., acetate, propionate, and butyrate) were separated on an HPLC-C_18_ column. The analytical method followed a previous publication (Hu et al., 2012). Each sample was detected in triplicate, and calibration curves based on the concentrations and peak areas of SCFA standards were used to assess the concentrations of individual SCFAs.

### Assessment of Jejunal Morphology

The jejunal samples were prepared according to Wang et al. (2016), and the fixed samples were then analyzed by Chengdu LiLai Biotechnology, Co., Ltd. Briefly, the fixed samples were embedded in paraffin, and approximately 4.50-μm sections were mounted on poly-Lys-coated slides, deparaffinized and rehydrated. The slides from each sample were stained with hematoxylin and eosin (HE) to examine morphology. Whole slide images were scanned by Mito More Than Microscopy, and 10 villi or crypts per sample were then selected to assess the intestinal architecture using the Motic Images Advanced 3.2 program (Motic China Group, Co., China). Then, the villus height: crypt-depth ratio (IVR) was calculated.

### Immunohistochemistry of the Proliferating Cell Nuclear Antigen (PCNA)

The immunohistochemistry protocol was adapted from a previously published method (Wang et al., 2015a). Briefly, paraffin-embedded tissue slides were first deparaffinized by soaking in 100% xylene and rehydrated with a decreasing percentage of ethanol. After the paraffin-embedded tissue slides were deparaffinized, they were incubated in a bath containing 10 mmol/L sodium citrate (pH 6.0) at 85–90°C for 45 min and then transferred to 0.20% sodium borohydride. Next, the slides were blocked in blocking buffer at room temperature for 45 min. Subsequently, the slides were incubated in porcine monoclonal-PCNA antibody (Bioss, Co., Beijing, China) overnight at 4°C. After the primary antibody was rinsed off, the slides were incubated with biotin-conjugated goat anti-pig immunoglobulins (1:200; Bioss, Co., Beijing, China) for 1 h at room temperature. Next, the slides were treated with horseradish peroxidase-conjugated streptavidin (ComWin Biotech, Co., Beijing, China) for 30 min and then reacted with 3,3-diaminobenzidine tetrahydrochloride (ComWin Biotech, Co., Beijing, China) as the colored chromogen to visualize the antigenic structures. The slides were then counterstained in Harris hematoxylin, mounted using liquid mounting medium with coverslips, and examined under bright field light microscopy at 100× magnification.

### RNA Extraction and Real-Time Reverse Transcription PCR

The RNA extraction and RT-qPCR protocols were adapted from a previously published method (Wang et al., 2018). Briefly, total RNA was extracted using Trizol (Invitrogen, Carlsbad, CA, United States) in accordance with the manufacturer’s protocol. The RNA concentration was measured using a NanoDrop ND-1000 spectrophotometer (Agilent Technologies, Palo Alto, CA, United States), and the purity of total RNA was then determined by the A260:280 and A260:230 ratios. For each sample, 1.00 μg of total RNA was reverse transcribed for complementary DNA (cDNA) synthesis using a PrimeScript^TM^ RT Reagent Kit with a gDNA Eraser (Takara Bio, Inc., Otsu, Japan) according to the manufacturer’s protocol. Real-time PCR reactions were performed in 96-well plates with a Bio-Rad CFX96TM Real-time PCR Detection System. The cycling program was 95°C (5 min) followed by 40 cycles of 95°C (10 s), 60°C (20 s), and 72°C (20 s). All reactions in this study were performed in triplicate for each cDNA sample.

### Selection of Reference and Target Genes and Primer Design

Five well-known porcine candidate reference genes and 14 target genes were selected using the GenBank database and according to a literature review of several of the reference genes (Supplementary Table S2). Prior to RT-qPCR, conventional PCR and agarose gel electrophoresis were performed to test the gene-specific primers and verify the amplified products. All of the PCR products were sequenced and then aligned against the pig genome with the BLAST program to verify their identity (Supplementary Tables S3, S4). The specificity of the primers was evaluated by melting curve analysis. Only those primers producing a single band of the expected size that had a sequence exactly matching a published sequence and that exhibited a unique peak during the dissociation step of the melting curve analysis were used for RT-qPCR.

### Determination of Digestive Enzyme Activities and Cytokines

The activities of alkaline phosphatase (AIP), creatine kinase (CK), and lactate dehydrogenase (LDH) in jejunal mucosa were assayed with Enzyme Activity Detection Kits (NanJing JianCheng Bioengineering Institute, Nanjing, China).

Additionally, the concentrations of porcine cytokines, including IL-1β, IL-8, TNF-α, and IFN-γ, were detected using commercially available ELISA kits (Quantikine Porcine Immunoassays, R&D Systems, Oxfordshire, United Kingdom). The dynamic assay range of IL-1β, IL-8, TNF-α, and IFN-γ was 39.10 to 2,500, 62.5 to 4,000, 23.40 to 1,500, and 39.00 to 2,500 pg/mL, respectively. The minimum detectable dose of IL-1β, IL-8, TNF-α, and IFN-γ was 13.60, 6.70, 5.00, and 11.20 pg/mL, respectively.

### Western Blotting Analysis

The procedure of western blotting was performed as described earlier (Li G. et al., 2014). Briefly, after protein samples were extracted from the jejunal mucosa, the protein contents were determined spectrophotometrically at OD_562_ using the Protein Quantitative Reagent Kit-BCA Method (ComWin Biotech, Co., Beijing, China). Primary antibodies of Bcl-2, Bax, activate-Caspase 3, activate-Caspase 9, GLP-2R, EGF, EGFR, ErbB, and B-actin were purchased from ABcam (Cambridge, United Kingdom), and primary antibodies of GRP41 and GRP43 were purchased from Santa Cruz Biotechnology (Santa Cruz, CA, United States). B-Actin was used as the loading control, and normalization and quantification of the bands was carried out using Quantity-One software (Bio-Rad).

### Statistical Analysis

For statistical differences in growth performances, the concentration of SCFAs, the levels and expression of pro-inflammatory cytokines, the expression of SCFA receptors, cell-proliferation markers and cell-apoptosis markers, the activities and expression of digestive enzymes, jejunal morphology, and the relative abundances of OTUs, we firstly confirmed that each sample at each time point is independent. Secondly, Shapiro–Wilk for normality test and Bartlett’s test for equal variances were then assessed, if the results were not accordance with both the normal distribution and equal variances, non-parametric analyses (Kruskal–Wallis test) were then applied in this study. Otherwise, the results would be assessed by one-way ANOVA and Duncan’s multiple comparison tests, with the different growth stage used as one factor. The SAS software (SAS Institute, Inc., Cary, NC, United States) was used for analysis, and differences were considered statistically significant at *P* < 0.05.

## Results

### Health and Growth of Piglets

During the 3-week post-weaning feeding trial, neither abnormal behavior nor diarrhea was observed (Table 1), suggesting that all piglets were healthy. BW, ADFI, and ADG increased with growth stage (post-weaning days 0–7 vs. 7–14 or 21) (*P* < 0.01), whereas the feed-to-gain ratio (F/G) showed no pronounced change (Table 1).

**Table 1:** The growth performance of weaned piglets.

	PW days 0–7	PW days 7–14	PW days 14–21	*P*-value	
			
	Means ± SEM				
ADG (g)^1^	184 ± 2.93^a^	333 ± 2.77^b^	396 ± 2.67^c^	<0.0001	
ADFI (g)^1^	285 ± 3.65^a^	507 ± 7.75^b^	642 ± 5.05^c^	<0.0001	
F/G^1^	1.55 ± 0.04	1.52 ± 0.03	1.62 ± 0.02	0.13	
Diarrhea score^2^	1.26 ± 0.03	1.10 ± 0.04	1.04 ± 0.04	0.46	

	**PW day 0**	**PW day 7**	**PW day 14**	**PW day 21**	***P*-value**
		
	**Means ± SEM**				

BW (kg)^2^	6.38 ± 0.01^a^	7.66 ± 0.02^b^	9.99 ± 0.02^c^	12.77 ± 0.01^d^	<0.0001


*PW, post-weaning; ADG, average daily gain; ADFI, average daily feed intake; F/G, feed-to-gain ratio; BW, body weight. ^*a,b,c*^Mean values within a row with different superscript letters are significantly different (*P* < 0.05). ^*1*^ADG, ADFI, and F/G (*n* = 5) and Diarrhea score (*n* = 40, 35, 30, 25 at PW days 0–7, 7–14, and 14–21). ^*2*^Diarrhea score and BW (*n* = 40, 35, 30, 25 at PW days 0, 7, 14, and 21).*

### Diversity and Population Dynamics of Post-weaning Jejunal Microbiota

#### DNA Sequence Data and Bacterial Community Structure

**FIGURE 1:**
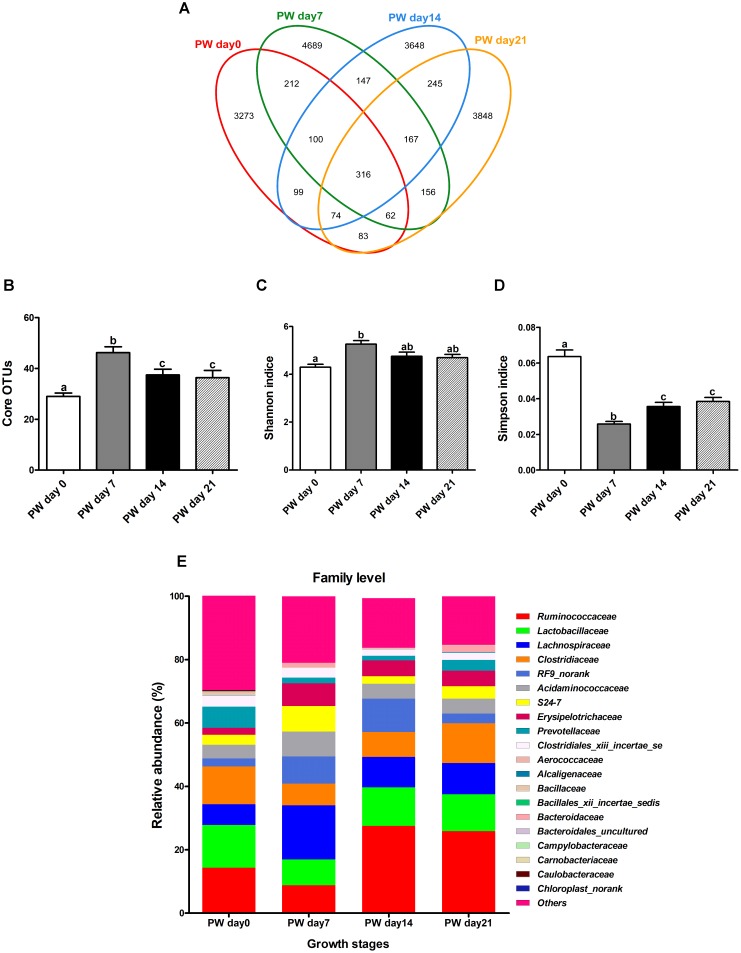
Jejunal microbial diversities and bacterial compositions of weaned piglets. Venn diagram showing the unique and shared operational taxonomic units (OTUs) (3% distance level) **(A)**, core OTUs (>1.00% of the community) **(B)**, estimated Shannon index **(C)**, estimated Simpson index **(D),** and bacterial compositions at the family level **(E)** of jejunal samples from weaned piglets. OTUs, operational taxonomic units; PW, post-weaning. Bars (mean ± SEM, *n* = 5) with different letters are considered significantly different (*P* < 0.05).

Across all twenty samples, 97,439 quality sequences were classified as being bacteria with an average read length 1,487-bp (Supplementary Table S5). The rarefaction curves generated by MOTHUR plotting the number of reads by the number of OTUs did not completely tended to approach the saturation plateau (Supplementary Figure S1). According to 97% species similarity, 4,219, 5,849, 4,769, and 4,951 OTUs were separately obtained from samples at days 0, 7, 14, and 21 post-weaning, respectively. Furthermore, 3,273, 4,689, 3,648, and 3,848 OTUs were uniquely identified at days 0, 7, 14, and 21 post-weaning, respectively (Figure 1A).

Large growth stage-related differences in core OTUs (>1.00% of the community) were observed (Figure 1B) along with alterations in alpha diversity (Figures 1C,D). Intriguingly, the number of core OTUs and Shannon indice increased throughout the first week post-weaning (*P* < 0.05) and largely stabilized from the second week post-weaning onwards, again with Simpson indice (*P* < 0.05) (Figures 1B–D).

To reveal the dissimilarity or similarity among samples, bacterial profiles from the jejunum were analyzed using the NMDS method. The profiles from the jejunum at post-weaning days 0, 7, 14, and 21 across horizontal space loosely clustered together in the NMDS plots for the respective days, with the profiles of post-weaning day 0 and day 21 being the farthest from the cluster center (Supplementary Figure S2). Supplementary Figure S3 shows a heatmap of jejunal microbiota at the genus level. The bacterial community profiles for post-weaning days 0, 7, 14, and 21 also grouped together with low similarity. Together, these results seem to suggest that the bacterial community profiles varied considerably with time after weaning.

LEfSe, an effect size measurement method, was then utilized to identify dominant OTUs. LDA (Supplementary Figure S4) showed that a total of 49 bacterial taxa (by day 28) differed in relative abundance (α = 0.01, LDA score > 3.0) among the tested groups. To further identify core OTUs, we employed probabilistic modeling using the LEfSe algorithm, which revealed 28, 5, 8, and 8 characteristic OTUs at post-weaning days 0, 7, 14, and 21, respectively (Supplementary Figure S4). Specifically, day 0 post-weaning indicator OTUs clustered into bacterial families *Clostridiales XIII incertae sedis*, *Erysipelotrichaceae*, *Verrucomicrobiaceae*, and *Porphyromonadaceae*, whereas post-weaning days 7, 14, and 21 indicator OTUs clustered into the main taxonomic groups, including *Lactobacillales, Ruminococcaceae, Pseudomonadaceae*, and *Peptostreptococcaceae*.

#### Population Dynamics at the Phylum Level

The OTUs (Supplementary Table S6) were classified into 13 bacterial phyla, although only the following four phyla had more than 1% overall relative abundance: *Firmicutes* (80.56%), *Bacteroidetes* (9.23%), *Tenericute*s (6.50%), and *Proteobacteria* (2.73%). Less than 1% of the sequences were not classified into bacterial phyla. In general, the relative abundances of these bacterial phyla underwent significant changes during the 3-week post-weaning period. Specifically, the trajectory from post-weaning days 0 to 21 was associated with an increasing relative abundance of *Firmicutes* (*P* < 0.01) while the relative abundances of *Bacteroidetes* (*P* < 0.01), *Tenericutes* (*P* < 0.01), and *Proteobacteria* (*P* < 0.01) decreased. Interestingly, the relative abundances of *Bacteroidetes* (*P* < 0.01) and *Tenericutes* (*P* < 0.01) at post-weaning day 7 differed from the corresponding abundances at the other time points.

#### Population Dynamics at the Class Level

At the class level (>5% overall relative abundance) (Supplementary Table S6), *Clostridia*, *Mollicutes*, *Bacteroidia*, *Negativicutes*, and *Bacilli* were the five most dominant classes during the post-weaning phase, accounting for 43.05, 15.58, 11.66, 7.96, and 7.20% of the total OTUs, respectively. Furthermore, the trajectory from post-weaning days 0 to 21 was associated with increasing relative abundances of *Clostridia* (*P* < 0.01) and *Bacilli* (*P* < 0.01) and decreasing relative abundances of *Mollicutes* (*P* < 0.01) and *Negativicutes* (*P* < 0.01). In addition, the relative abundances of *Clostridia* and *Bacilli* increased during the first week (*P* < 0.01) and then largely stabilized from the second week post-weaning onwards (*P* > 0.05).

#### Population Dynamics at Family Level

At the family level (Figure 1E and Supplementary Table S6), the most dominant families (>4.50% overall relative abundance) during the post-weaning phase were *Ruminococcaceae* (19.14%), *Lactobacillaceae* (11.36%), *Lachnospiraceae* (10.75%), *Clostridiaceae* (9.82%), *RF9_norank* (6.15%), *Acidaminococcaceae* (5.39%), *Erysipelotrichaceae* (4.80%), and *S24-7* (4.39%).

**FIGURE 2:**
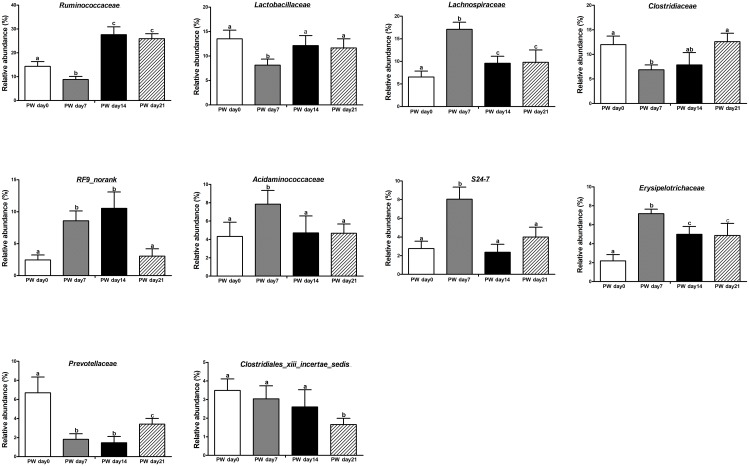
The core bacteria (at the family level) in the jejunum of weaned piglets. PW, post-weaning. Bars (mean ± SEM, *n* = 5) with different letters are considered significantly different (*P* < 0.05).

Post-weaning changes in the core dominant families (Figure 2) included increasing relative abundances of *Lachnospiraceae* (*P* < 0.01), *Erysipelotrichaceae* (*P* < 0.01), and *RF9_norank* (*P* < 0.05) and decreasing relative abundances of *Lactobacillaceae* (*P* < 0.05), *Ruminococcaceae* (*P* < 0.01), *Acidaminococcaceae* (*P* < 0.05), and *S24-7* (*P* < 0.01). Notably, the relative abundances of *Lachnospiraceae*, *Erysipelotrichaceae*, and *Acidaminococcaceae* increased over the first week post-weaning (*P* < 0.01) and largely stabilized from the second week post-weaning onwards (*P* > 0.05), again with *Lactobacillaceae* and *Ruminococcaceae* (*P* < 0.05).

The most common core OTUs (>2.00% relative abundance in ≥80.00% of piglets) are listed in Supplementary Table S7. At the family level, 7, 9, 7, and 9 core OTUs with >2% relative abundance were identified at 0, 7, 14, and 21 days post-weaning, respectively. Four core OTUs were present throughout the entire post-weaning phase: *Ruminococcaceae*, *Lactobacillaceae*, *Lachnospiraceae*, and *Clostridiaceae*. However, *Erysipelotrichaceae* was only family that did not present at post-weaning day 0.

### SCFA Concentrations and Receptor Expression

**FIGURE 3:**
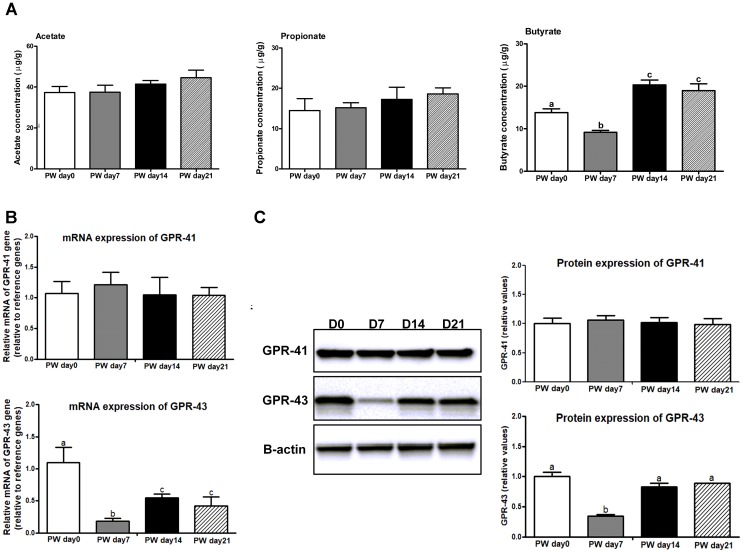
The SCFAs and its receptors in the jejunum of weaned piglets. **(A)** The ratios of acetate, propionate, and butyrate to total SCFAs in the jejunum of piglets during the first 3 weeks post-weaning. **(B)** The mRNA expression of SCFAs receptors (e.g., GPR41 and GPR43) in the jejunum of weaned piglets. **(C)** Representative western blot and quantification of SCFAs receptors (e.g., GPR41 and GPR43) in the jejunum of weaned piglets. B-Actin was used as the loading control. SCFAs, short-chain fatty acids; PW, post-weaning; D0, post-weaning day 0; D7, post-weaning day 7; D14 = post-weaning day 14; D21, post-weaning day 21. Bars (mean ± SEM, *n* = 5) with different letters are considered significantly different (*P* < 0.05).

The jejunal butyrate concentration at post-weaning day 7 was lower (*P* < 0.05) than at post-weaning day 0; it was also lower at post-weaning day 14 (*P* < 0.01) and 21 (*P* < 0.01) (Figure 3A). However, the concentrations of acetate and propionate did not change during the 3-week post-weaning period.

We further investigated whether decreased butyrate inhibited the expression of the SCFA receptors GPR-41 and GPR-43 (Figures 3B,C). The mRNA and protein expression of GPR-43 were decreased (*P* < 0.01) at post-weaning day 7 relative to the levels at post-weaning days 0, 14 and 21, whereas no difference in GPR-41 expression among time points was observed.

### Correlation Between Microbial Community Composition and SCFAs

Next, a Spearman’s correlation test was used for assessing the relationships between the core bacteria at family level and SCFAs. As revealed in Table 2, butyrate was negatively associated with the relative abundances of *Lachnospiraceae* (Spearman ρ = -0.71, *P* < 0.01) and *Erysipelotrichaceae* (Spearman ρ = -0.62, *P* < 0.01) but positively correlated with the relative abundances of *Lactobacillaceae* (Spearman ρ = 0.53, *P* < 0.001) and *Ruminococcaceae* (Spearman ρ = 0.70, *P* = 0.02). No positive or negative correlation was found between either of the other two SCFAs (i.e., acetate and propionate) and the relative abundances of the core bacteria.

**Table 2:** Spearman’s correlation coefficients and relative *P*-values between the relative abundances of core bacteria (at the family level) and SCFAs production in the jejunum of weaned piglets.

	Acetate		Propionate		Butyrate	
			
	Correlation coefficient	*P*-value	Correlation coefficient	*P*-value	Correlation coefficient	*P*-value
*Ruminococcaceae*	-0.14	0.55	-0.45*	0.05	0.70**	<0.01
*Lactobacillaceae*	0.06	0.81	-0.29	0.43	0.53*	0.02
*Lachnospiraceae*	-0.63	0.79	0.50	0.16	-0.71**	<0.01
*Clostridiaceae*	0.10	0.69	-0.04	0.89	0.11	0.64
*RF9_norank*	0.14	0.54	0.08	0.75	-0.39	0.09
*Acidaminococcaceae*	-0.16	0.51	0.32	0.17	-0.34	0.14
*S24-7*	-0.31	0.18	0.25	0.30	-0.49	0.03
*Erysipelotrichaceae*	0.11	0.65	0.49*	0.01	-0.62**	<0.01
*Prevotellaceae*	0.17	0.47	-0.11	0.65	0.06	0.82
*Clostridiales_xiii_incertaesedis*	-0.18	0.48	0.05	0.85	-0.30	0.20


*The correlation is expressed with the r coefficient. ^∗^*P* < 0.05; ^∗∗^*P <* 0.01.*

### Pro-inflammatory Cytokines

**FIGURE 4:**
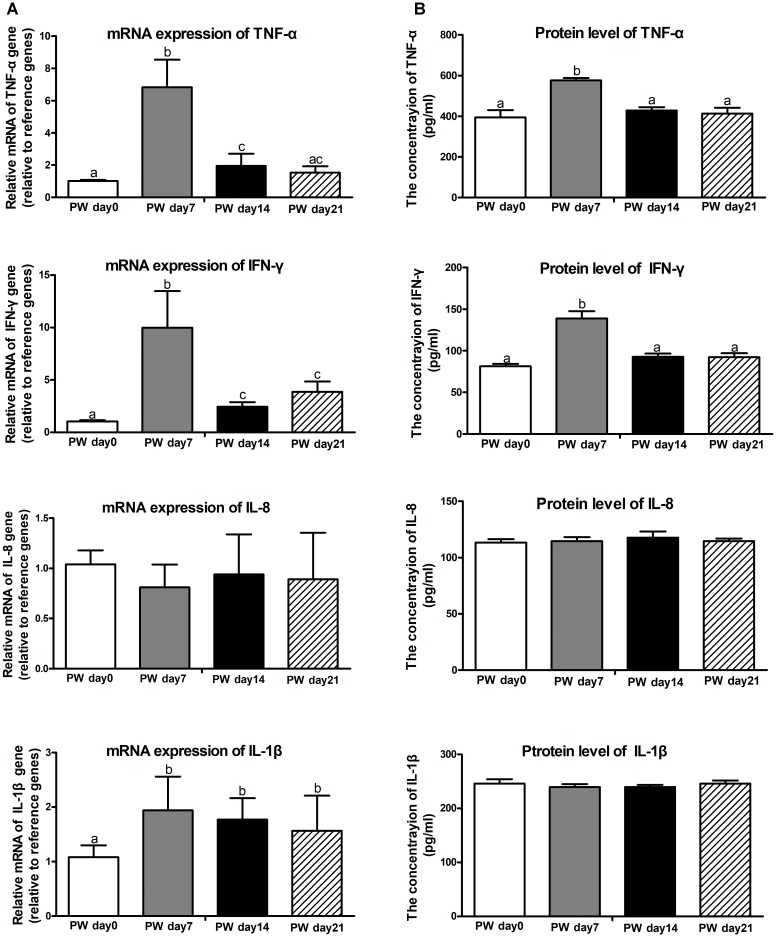
Pro-inflammatory cytokines in the jejunum of weaned piglets. The mRNA expression **(A)** and protein levels **(B)** of pro-inflammatory cytokines (i.e., TNF-α, IFN-γ, IL-8, and IL-1β). PW, post-weaning. Bars (mean ± SEM, *n* = 5) with different letters are considered significantly different (*P* < 0.05).

Next, we investigated whether butyrate is potentially associated with the regulation of pro-inflammatory cytokines. The mRNA (Figure 4A) and protein expression (Figure 4B) of TNF-α (*P* < 0.01) and IFN-γ (*P* < 0.01) were increased at post-weaning day 7 relative to the corresponding levels at the other time points (i.e., post-weaning days 0, 14, and 21), whereas no changes in expression among all time points were observed for IL-1β and IL-8.

### Cell Proliferation

**FIGURE 5:**
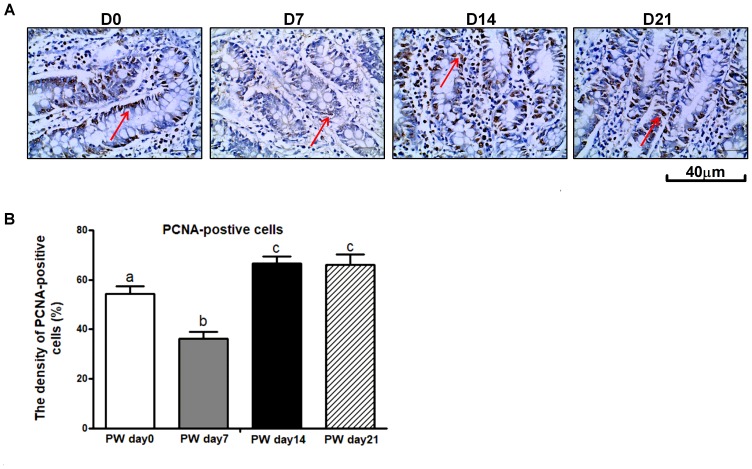
The proliferating cell nuclear antigen (PCNA) in the jejunum of weaned piglets. **(A)** Representative images used for the immunohistochemical detection of PCNA in the jejunum segment of piglets at post-weaning days 0, 7, 14, and 21. **(B)** The density of PCNA-positive cells in the jejunum of piglets at post-weaning days 0, 7, 14, and 21. PWD, post-weaning day; D0, post-weaning day 0; D7, post-weaning day 7; D14, post-weaning day 14; D21, post-weaning day 21. Bars (mean ± SEM, *n* = 5) with different letters are considered significantly different (*P* < 0.05).

**FIGURE 6:**
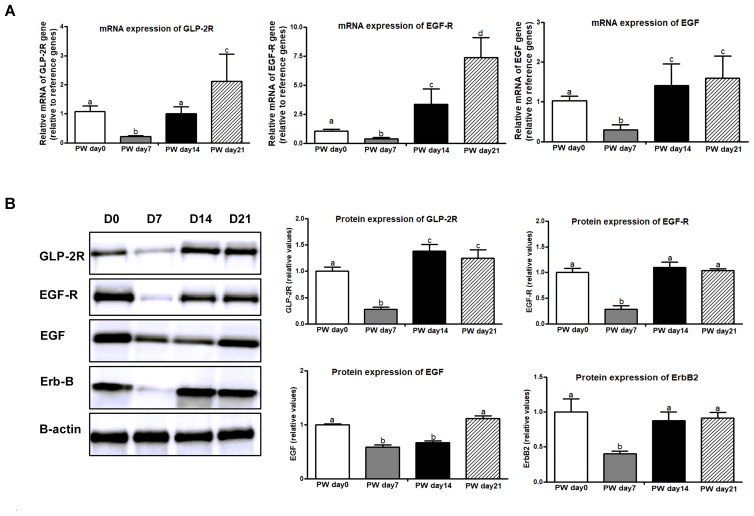
The signaling markers of cell proliferation along the jejunal crypt-villus axis of weaned piglets. **(A)** The mRNA expression of cell-proliferation makers (e.g., GLP-2R, EGF-R, and EGF). **(B)** Representative western blot and quantification of cell-proliferation markers (e.g., GLP-2R, EGF-R, EGF, and ErbB). B-Actin was used as the loading control. PWD, post-weaning day; D0, post-weaning day 0; D7, post-weaning day 7; D14, post-weaning day 14; D21, post-weaning day 21. Bars (mean ± SEM, *n* = 5) with different letters are considered significantly different (*P* < 0.05).

One of the possible mechanisms whereby weaning stress affects intestinal morphology is the stimulation of intestinal cell proliferation (Cheung et al., 2009). PCNA is an auxiliary protein of DNA polymerases δ and ε-enzymes required for DNA synthesis. It is highly expressed at the S-phase of the cell cycle and has therefore been used as an indicator of cell proliferation (Muskhelishvili et al., 2003). To examine the enterocyte proliferation status during the 3-week post-weaning period, immunohistochemical analyses were performed on paraffin-embedded intestinal tissue sections by using antibody against PCNA. As shown in Figures 5A,B, the percentage of crypt cells bearing PCNA-positive nuclei at post-weaning days 0, 7, 14, and 21 was approximately 54, 36, 67, and 65%, respectively. Consequently, a lower intensity of cells positive for PCNA was observed at post-weaning day 7 than at post-weaning days 0, 14, and 21 (*P* < 0.01). In investigating the cell proliferative system along the crypt-villus axis, we found that mRNA (Figure 6A) and protein expression (Figure 6B) of signaling markers (i.e., ErbB, GLP-2R, EGF, and EGFR) was decreased at post-weaning day 7 relative to expression at post-weaning day 0 (*P* < 0.05) and that the trajectory from post-weaning days 7 to 21 was associated with increasing mRNA and protein expression of ErbB, GLP-2R, EGF, and EGFR (*P* < 0.05).

### Cell Apoptosis

**FIGURE 7:**
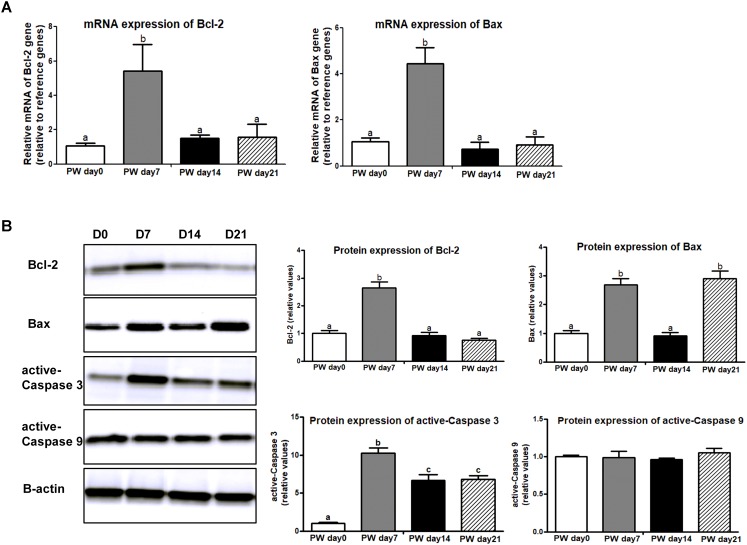
The signaling markers of cell apoptosis along the jejunal crypt-villus axis of weaned piglets. **(A)** The mRNA expression of cell-apoptosis makers (e.g., Bcl-2 and Bax). **(B)** Representative western blot and quantification of cell-apoptosis makers (e.g., Bcl-2, Bax, active-Caspase 3, and active-Caspase 9). B-Actin was used as the loading control. PWD, post-weaning day; D0, post-weaning day 0; D7, post-weaning day 7; D14, post-weaning day 14; D21, post-weaning day 21. Bars (mean ± SEM, *n* = 5) with different letters are considered significantly different (*P* < 0.05).

Apoptosis is a major form of programmed cell death that is regulated by the Bcl-2 family (i.e., Bcl-2 and Bax) and caspase family (i.e., active-Caspase 3 and active-Caspase 9). In this study, mRNA expression of Bcl-2 and Bax was higher at post-weaning day 7 than at the other growth stages (*P* < 0.01), and the trajectory from post-weaning days 7 to 21 was associated with decreasing mRNA expression of Bcl-2 and Bax (*P* < 0.01) (Figure 7A). A similar trend was observed in protein expression of Bax and active-Caspase 3 (*P* < 0.01), but no significant change over time was shown in activate-Caspase 9 (Figure 7B).

### Jejunal Morphology

**FIGURE 8:**
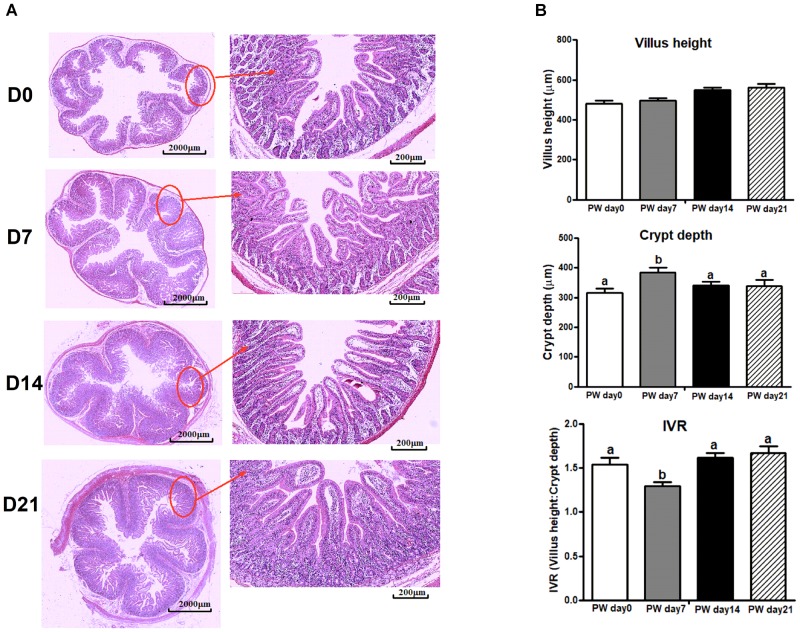
The jejunal morphology of weaned piglets. **(A)** Representative light micrographs of a cross-section of the jejunum of piglets at post-weaning days 0, 7, 14, and 21. Images of the whole slide were scanned by Mito More Than Microscopy, and images were then taken at 40× magnification using a light microscope. **(B)** The quantification of villus height, crypt-depth and the ratio of villus height/crypt-depth (IVR). PWD, post-weaning day; D0, post-weaning day 0; D7, post-weaning day 7; D14, post-weaning day 14; D21, post-weaning day 21. Bars (mean ± SEM, *n* = 5) with different letters are considered significantly different (*P* < 0.05).

Jejunal morphology [i.e., villus height, crypt-depth, and villus height/crypt-depth (IVR)] is shown in Figure 8. There were no significant changes in villus height over the 3-week post-weaning period, but increased crypt-depth (*P* < 0.05) and decreased IVR (*P* < 0.05) were observed at post-weaning day 7. Notably, the trajectory from post-weaning days 7 to 14 was associated with a decrease in crypt-depth (*P* < 0.05) and an increase in IVR (*P* < 0.05).

### Digestive Enzymes

**FIGURE 9:**
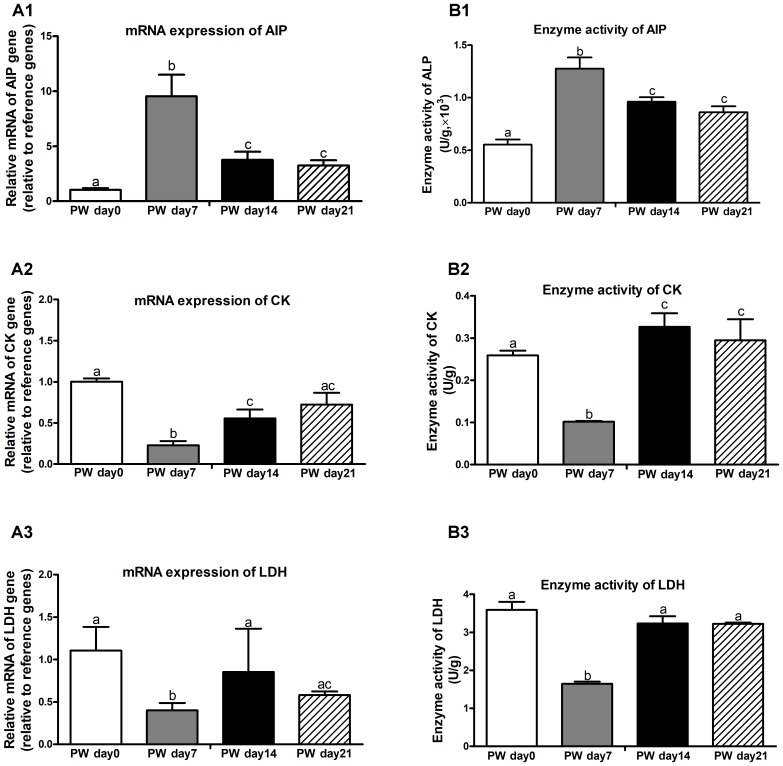
Digestive enzymes in the jejunum of weaned piglets during the first 3 weeks post-weaning. The mRNA expression **(A)** and activities **(B)** of digestive enzymes (e.g., AIP, CK, and LDH). PW, post-weaning; AIP, alkaline phosphatase; CK, creatine kinase; LDH, lactate dehydrogenase. Bars (mean ± SEM, *n* = 5) with different letters are considered significantly different (*P* < 0.05).

Brush border enzymes, such as AIP, CK, and LDH, are commonly used as cell markers of villus maturation, and their activities serve as markers of intestine damage (Wang et al., 2016). The mRNA expression and activities of digestive-enzyme markers (i.e., AIP, CK, and LDH) are presented in Figure 9. During the 3-week post-weaning period, the highest mRNA expression (Figure 9A) and activities (Figure 9B) of AIP (*P* < 0.01) and the lowest mRNA expression and activities of CK (*P* < 0.01) and LDH (*P* < 0.05) were found at post-weaning day 7.

## Discussion

**FIGURE 10:**
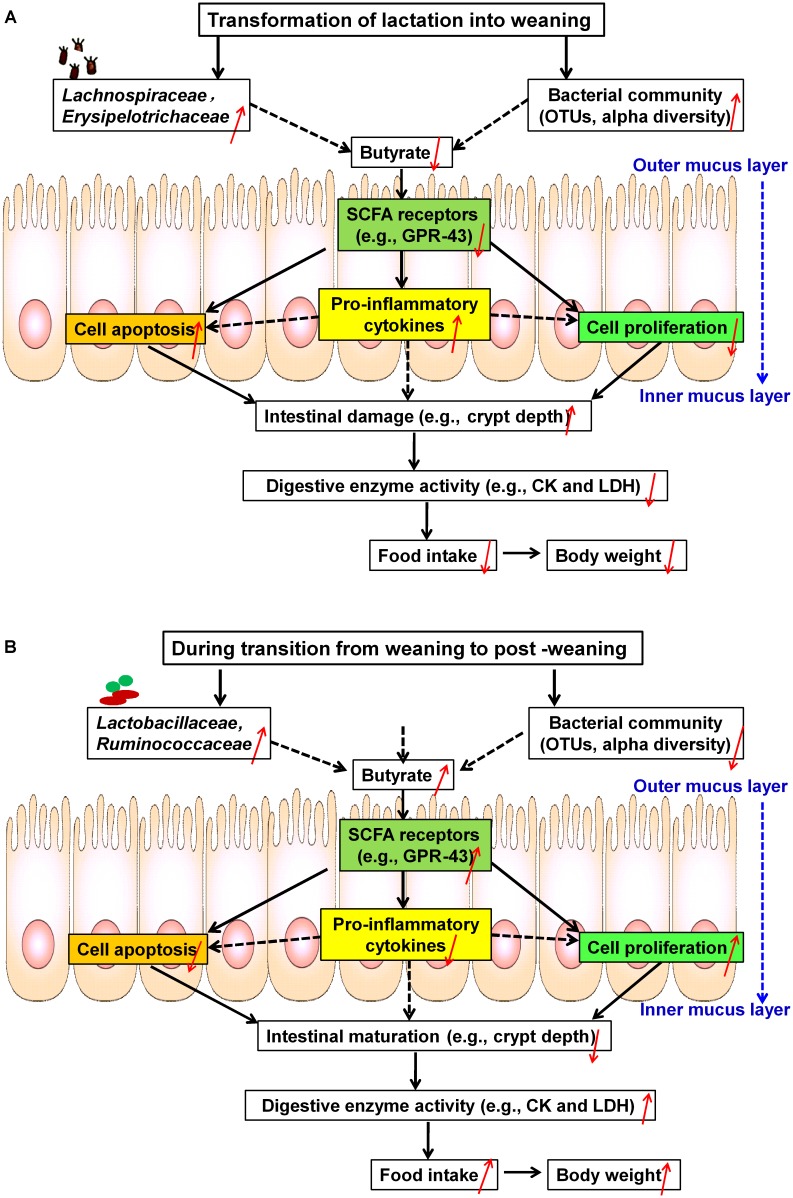
Schematic representation of microbial metabolite butyrate-induced intestinal adaptation via the immune response to weaning stress. **(A)** Weaning stress-induced intestinal dysfunction: at the first week post-weaning (transition from lactation to weaning), weaning stress induces significant alterations of core OTUs and alpha diversity, especially increases in two dominant taxonomic groups (i.e., *Erysipelotrichaceae* and *Lachnospiraceae*). These changes result in decreased butyrate production and decreased expression of its receptor (GPR-43), which contributes to the onset of apoptosis and the inhibition of proliferation along the crypt-villus axis via inducing increased pro-inflammatory cytokines. As a result, pronounced shifts of the intestinal structure and function toward post-weaning intestinal dysfunction (increased crypt-depth) occur, which significantly decrease digestive-enzyme activities (i.e., CK and LDH) and growth check (i.e., low feed intake and body weight). **(B)** Self-repairing mechanism-induced jejunal adaptation toward health status: at the second week post-weaning (transition from weaning to post-weaning), increasing the relative abundances of *Lactobacillaceae* and *Ruminococcaceae* are accompanied by decreased in core OTUs and the alpha diversity of bacteria community. These changes stimulate butyrate production and expression of its receptor, which lead to the inhibition of apoptosis and the stimulation of proliferation via decreased pro-inflammatory cytokines, thereby promoting the development of post-weaning intestinal environment toward health status. OTUs, operational taxonomic units; CK, creatine kinase; LDH, lactate dehydrogenase.

The results led us to formulate the two following conclusions: (1) During the first week after weaning (Figure 10A), significant increases were observed in core OTUs and the alpha diversity of the bacterial community, particularly in the relative abundances of the two dominant bacterial families (i.e., *Erysipelotrichaceae* and *Lachnospiraceae*). These OTU and diversity increases in *Erysipelotrichaceae* and *Lachnospiraceae* were associated with decreased butyrate production and decreased expression of its receptor (GPR43), which induced the onset of apoptosis and the inhibition of proliferation via increased pro-inflammatory cytokines. Furthermore, the apoptosis/proliferation imbalance resulted in crypt elongation and villous atrophy, thereby decreasing digestive-enzyme activities and growth check. (2) During the second week after weaning (Figure 10B), the relative abundances of *Lactobacillaceae* and *Ruminococcaceae* were accompanied with increased butyrate production and expression of its receptor. These increases led to decreased cell apoptosis and increased cell proliferation via decreased pro-inflammatory cytokines and therefore to the recovery of intestinal morphology and the improvement of digestive-enzyme activities and growth performance. Together, the results indicate that microbial-driven butyrate might be a key modulator in the maintenance of jejunal homeostasis after weaning. Each of these aspects will be discussed in more detail below.

### Weaning Is a Key Driver of Shifts in the Microbial Community of the Jejunum

The current result indicated that the jejunal environment experienced a significant turnover in microbial community composition and structure at the first week post-weaning and that the composition and structure largely stabilized at 2–3 weeks post-weaning. Our observation was consistent with the results of previous studies in weaned piglets (McLamb et al., 2013; Slifierz et al., 2015; Hu et al., 2016). For example, a significant increase was observed in total OTUs and Shannon indices of fecal samples from weaned piglets at the first week post-weaning (Slifierz et al., 2015). Additionally, alpha diversity of bacterial community in Congjiang-miniature piglets after weaning also experienced a remarkable declined, and its gut microbiota was then shifted dramatically (Hu et al., 2016). Consequently, the current results further support previous findings that the weaning process is a key driver of shifts in the gut microbial community in piglets (Merrifield et al., 2013; Hu et al., 2016).

In addition to the changes in intestinal microbial community, we also found that both of feed intake and BW dramatically decreased at the first week post-weaning. In swine industry, besides other additional stressors, including some sudden changes in the social (i.e., mixing with unfamiliar piglets) and physical environments (i.e., transferring to an unfamiliar pen), the piglets at weaning mainly suffer from the abrupt separation from the sows, and subsequently a significant change in diet (van der Meulen et al., 2010). As a result, dramatic decreases in feed intake at the first week post-weaning cannot meet the energy requirement and metabolism (van Beers-Schreurs and Bruininx, 2002), and the intestinal microbiota is then significant shifted toward later-on pathogen shedding and disease susceptibility (Slifierz et al., 2015; Hu et al., 2016). Unlike infants, the weaning process in piglets is a rapid dietary shift (Frese et al., 2015). By the end of the first week post-weaning, metabolizable energy intake accounts for approximately 60.00–70.00% of pre-weaning milk intake and that it takes around 2 weeks post-weaning to fully recover its pre-weaning level (Campbell et al., 2013). Regardless of weaning age, the piglets in general will lose approximately 100–250 g BW at the first day post-weaning, and then recover this loss of BW until the end of the first week post-weaning (Le Dividich and Seve, 2000). Even of it is very tough for preventing the decline of BW as the piglets transfer from sow milk intake to the solid diet (Kojima et al., 2007), to rapid maintain or improve weaned-piglets eating and growing would be an effective strategy to decrease challenges and impacts of low feed intake on its intestinal microbial population ecology (Campbell et al., 2013).

### Regulation of Inflammatory Response by Butyrate-Producing Bacteria in the Jejunum

In this study, significant increases in TNF-α and IFN-γ were accompanied by marked decreases in jejunal butyrate production. Butyrate, as a main end-product of intestinal microbial fermentation, was closely related to the immune system in the GIT (Meijer et al., 2010). A Spearman’s correlation test was further used for investigating the relationships between butyrate production and core bacterial families, we found that butyrate production was negatively correlated with the relative abundances of *Erysipelotrichaceae* and *Lachnospiraceae* but positively correlated with the relative abundances of *Lactobacillaceae* and *Ruminococcaceae.*

In the early post-weaning period, the balance between the development of so-called healthy commensal microbiota and the establishment of bacterial intestinal disease can be easily tipped toward disease expression (Hopwood and Hampson, 2003). Here, we found that *Erysipelotrichaceae* significantly increased at the first week post-weaning. Associations between *Erysipelotrichaceae* and pro-inflammatory cytokines (i.e., TNF-α) was also found previously (Dinh et al., 2015). Recent reports further documented a potential role of *Erysipelotrichaceae* in host physiology and/or inflammation-related disorders in the GIT (Kaakoush, 2015). *Lachnospiraceae*, as the dominant bacteria, was also increased at the first week post-weaning in this study. Previous reports demonstrated that rotavirus infection led to increased levels of *Lachnospiraceae* and increased mRNA expression of IFN-γ, IL-8, and IL-10 in the ileum of pigs (Li M. et al., 2014). Considering the results together, we hypothesize that the increases of *Lachnospiraceae* and *Erysipelotrichaceae* after weaning potentially influence host inflammatory responses.

Additionally, the relative abundances of the other two dominant families (*Lactobacillaceae* and *Ruminococcaceae*) significantly increased at 2 weeks post-weaning in this study. Some evidences already reported that *Lactobacillaceae* and *Ruminococcaceae* increased with age in piglets (Bäckhed et al., 2015; Slifierz et al., 2015). For *Lactobacillus*, it is classified under *Lactobacillaceae* and belongs to the lactic acid bacteria that is able to effectively enhance the innate and adaptive immunity of the host, and an increase in *Lactobacillaceae* potentially triggers the host immune response (Ma et al., 2017). As a common inhabitant of the mucosal surface in the gut, *Lactobacillaceae* is of particular interest with respect to protection of the gut against potential pathogens (e.g., *Prevotella* and *Haemophilus parasuis*) by preventing colonization (Zhang et al., 2016). Recent studies demonstrated that *Lactobacillus* supplements could maintain the physiological balance and stimulate the growth of piglets (Kim and Isaacson, 2015). As SCFAs-producing bacteria, *Ruminococcaceae* is also commonly found in the intestine of mammals and displays the ability to degrade hemicellulose and cellulose of plant material in solid diet (Jiang et al., 2015). Meanwhile, a metagenome prediction analysis also indicated that *Ruminococcaceae* indirectly contributed to butyrate production (Esquivel-Elizondo et al., 2017). In neonatal piglets, the increase in the relative abundance of *Ruminococcaceae* was accompanied by the decreases in the expression of pro-inflammatory genes (i.e., IL-6, IL-8, and IFN-γ) and anti-inflammatory genes (i.e., IL-10 and TGF-β) in the ileum of piglets that fed with sodium butyrate (Xu et al., 2016). In response to an infectious challenge after weaning, the primary goal of the innate immune system is to rapidly clear offending pathogens to prevent prolonged inflammation and sepsis (McLamb et al., 2013), and the increase of *Lactobacillaceae* and *Ruminococcaceae* might be a potential beneficial component of the inflammatory response to weaning stress in piglets, however, it still need to be further studied.

### The Cross Talk Among Apoptosis, Proliferation, and Inflammatory Response

Butyrate seems to exert broad anti-inflammatory activities by modulating immune cell migration, adhesion, cytokine expression, cell proliferation and cell apoptosis, and the effects of butyrate on the immune system are mediated by GPRs, in particular GPR41 and GPR43, which are both highly expressed on immune cells (Meijer et al., 2010). From the perspective of SCFAs, acetate preferentially activates GPR43, propionate displays similar agonism on both of GPR41 and GPR43, and butyrate preferentially activates GPR41 and GPR109A (Lin et al., 2012). On the contrary, an association between butyrate production and GPR-43 expression was observed in this study. Possible immune-modulatory functions of SCFAs, especially butyrate, were highlighted in a recent study in GPR43-/- mice, it indicated that GPR43 binding of SCFAs potentially provided a molecular link among diet, gastrointestinal bacterial metabolism, and inflammatory responses (Maslowski et al., 2009). Consistent with previous findings, the current study also found that a significant decrease in GRP43 expression was accompanied by the increases in pro-inflammatory cytokines during the first 3 weeks after weaning. Unlike the sucking period, a possible reason might be that the piglets suffered from the high level of pathogenic stress during the weaning process, and decreased butyrate production then contributes to intestinal inflammation via activating GPR43 expression. Actually, the present study was lack of the independent validation of the data generated. For weaned piglets, to fully understand this mechanism with respect to the associations between butyrate and GPR43, both of *in vitro* and *in vivo* pathogenic challenge models that supplemented with butyrate is still needed in further studies.

A precise balance between cell proliferation and apoptosis, which is regulated by cell cycle regulators, is required for gut homeostasis (Zhu et al., 2014). However, a decrease in immune function as induced by weaning stress can stimulate apoptosis and inhibit cell proliferation along the crypt-villus axis, thereby resulting in intestinal dysfunction and atrophy (Campbell et al., 2013). A previous study further demonstrated that weaning stress was a major trigger of intestinal cell apoptosis (Zhu et al., 2014). Consistent with these findings, we also found that the upregulation of cell apoptosis genes (i.e., Bcl-2, Bax, and active-Caspase 3) at the first week post-weaning. As a pro-apoptotic member of the Bcl-2 family, cell death antagonist (Bcl-2) regulates apoptotic mitochondrial events through the regulation of cytochrome C release from the mitochondria via the alteration of mitochondrial membrane permeability (Zha et al., 1996; Zhu et al., 2014). At weaning, the distribution of apoptosis in the intestinal crypt cells is associated with anti-apoptotic members of the Bcl-2 family, and the Bcl-2/Bax is an apoptotic signal for intestinal inflammation that contributes to intestinal dysfunction and atrophy (Merritt et al., 1995; Hawkins and Vaux, 1997).

Moreover, significant decreases were also observed in mRNA and protein expression of cell proliferation markers (i.e., GLP-2R, ErbB, EGFR, and EGF) at the first week post-weaning, whereas increases were found at 2 weeks post-weaning. For the proliferation pathway in intestinal crypt cells, GLP-2R, expressed by intestinal subepithelial myofibroblasts (ISEMFs) and enteroendocrine cells, acts via GLP-2R binding on ISEMF cells, which leads to the release of ISEMF-derived ErbB-signaling network ligands, including EGF, betacellulin, epigen, TGF-α, epiregulin, amphiregulin, and heparin-binding-EGF (Rowland and Brubaker, 2011). EGF then binds to the EGF-R located on crypt epithelial cells, where it transactivates the ErbB receptor, stimulating cell proliferative responses when local inflammatory cytokine patterns are altered (Yusta et al., 2009). The disruption of ErbB signaling, induced by the alteration of local inflammatory cytokine patterns, is associated with the disturbed development of the intestinal mucosa (Yusta et al., 2009; Rowland and Brubaker, 2011). Consequently, it seems that 2–3 weeks post-weaning might be a key period for immune maturation involving the regulation of cell apoptosis and proliferation.

As intestinal inflammation in response to weaning stress contributes to alterations in digestive-enzyme activities (i.e., AIP) (Lackeyram et al., 2010), and is related to the permeability and transport of nutrients, altered local inflammatory cytokine patterns along the crypt-villus axis after weaning have negative influences on intestinal integrity and epithelial function (McKay and Baird, 1999; Campbell et al., 2013). Weaning-associated gut mucosal atrophy involves variable expression patterns of intestinal digestive enzymes, such as decreases in LDH and CK activities (Wang et al., 2016) as well as increases in AIP activity (Lackeyram et al., 2010). These three digestive enzymes are commonly regarded as key enzyme markers when considering changes in the primary digestive and absorptive functions of the small intestine (Lackeyram et al., 2010; Wang et al., 2016). Similar to previous observations in weaned piglets, this study further found that weaning stress induced decreased LDH and CK activities but increased AIP activity.

Consistent with the finding of Boudry et al. (2004), the current findings further suggest that weaning induced the maturation of the immune system and was a key period of maturation of the physiological functions in the jejunum. Although the development of pro-inflammatory cytokines in the jejunum of weaned piglets was compiled with its age in this study, we did not investigate whether this change was mainly triggered by different weaning ages or a rapid dietary shift, and therefore the two factors would be considered in our further studies. Finally, the individual rarefaction curves of all samples did not completely tend to approach the saturation plateau, it seems that this sequencing depth was not enough to cover the whole bacterial diversity in this study. The present characterization of the intestinal microbiota of conventionally-raised animals using the next-generation sequencing is only representative of one of hypervariable regions (V1–V9), and complete 16S rRNA sequence may be absent so far. Although the complete 16S rRNA sequence give a more accurate measure of taxonomic diversity, the increase of sequencing depth is also needed on the PacBio^®^ RS II platform.

## Conclusion

During the transition from lactation to weaning, increases in the relative abundances of *Lachnospiraceae* and *Erysipelotrichaceae* inhibited butyrate production and its receptor expression, resulting in an unbalance of apoptosis/proliferation. This inhibition was most likely due to increased pro-inflammatory cytokines and resulted in jejunal adaptation toward intestinal dysfunction. However, during the transition from weaning to post-weaning, increasing the relative abundances of *Lactobacillaceae* and *Ruminococcaceae* increased butyrate production, stimulating jejunal adaption toward gut health. Therefore, for weaned piglets, microbial-driven butyrate might be a key modulator of the balance of apoptosis/proliferation via the regulation of inflammatory responses, thereby stimulating its intestinal development and later growth. These findings potentially inform the development of approaches for increasing butyrate and inhibiting intestinal inflammation and thereby improving jejunal adaptation toward gut health during the weaning process.

## Availability of Data and Materials

The dataset supporting the results of this article has been deposited in the NCBI Sequence Read Archive under BioProject PRJNA454828.

## Author Contributions

XZ, SW, and ZZ designed the experiments. XZ, SW, ZZ, AS, and MN wrote and revised the manuscript. XZ, SW, LC, LZ, and ZDZ performed the experiments and analyzed the results. ZZ and SW supervised and coordinated the project. All authors read and approved the final manuscript.

## Conflict of Interest Statement

LZ and ZDZ was employed by Shenzhen Premix Inve Nutrition, Co., Ltd. and Chengdu LiLai Biotechnology, Co., Ltd., respectively. The remaining authors declare that the research was conducted in the absence of any commercial or financial relationships that could be construed as a potential conflict of interest.

## Footnotes

1 http://drive5.com/uparse/

2 http://www.arb-silva.de

3 http://www.mothur.org

4 https://www.mothur.org/wiki/Chao

5 https://www.mothur.org/wiki/Ace

6 https://www.mothur.org/wiki/Simpsonsimilar

7 https://www.mothur.org/wiki/Shannon

8 http://huttenhower.sph.harvard.edu/galaxy/
