# Supplementary material for: Microbial-Driven Butyrate Regulates Jejunal Homeostasis in Piglets During the Weaning Stage
Source: Front Microbiol. 2019 Jan 18;9:3335. doi: 10.3389/fmicb.2018.03335 (PMC6345722; doi:10.3389/fmicb.2018.03335)
Supplement: Supplementary file 1 [file Data_Sheet_1.docx]

**Additional file 1**

**TABLE S1. Composition and nutrient levels of the basal diet (as fed-basis).**

| Item | Content, % |
| --- | --- |
| Ingredients |  |
| Corn (CP^1^ 8.70%) | 55.94 |
| Wheat | 5.50 |
| Whey, dried | 5.70 |
| Soybean meal | 10.82 |
| Soybean protein (CP^1^ 65.00%) | 12.65 |
| Soybean oil | 1.60 |
| Fishmeal | 3.50 |
| L-Lysine-HCl | 0.12 |
| DL-Methionine | 0.11 |
| L-Threonine | 0.05 |
| Sugar | 1.50 |
| Limestone | 0.80 |
| Salt | 0.26 |
| Dicalcium phosphate | 0.55 |
| Vitamin premix^2^ | 0.40 |
| Trace mineral premix^3^ | 0.60 |
| Total | 100.00 |
| Energy and nutrient composition^4^ |  |
| ME^5^, kcal/kg | 3,420 |
| CP^4^, % | 22.80 |
| Crude fat, % | 4.40 |
| Lys, % | 1.42 |
| Met, % | 0.52 |
| Met + Cys, % | 0.85 |

^1^Vitamins were provided in the following amounts per kilogram of the diet: vitamin A, 10,000 IU; vitamin D_3_, 1,500 IU; vitamin E, 50 IU; vitamin K_3_, 2.50 mg; vitamin B_12_, 60 μg; vitamin B_1_, 4.50 mg; vitamin B_2_, 12 mg; niacin, 60 mg; pantothenic acid, 36 mg; folic acid, 1 mg; vitamin B_6_, 10 mg; biotin, 0.50 mg; and vitamin C, 200 mg.

^2^Trace minerals were provided in the following amounts per kilogram of the diet: Fe, 100 mg; Cu, 6 mg; Mn, 4 mg; Zn, 100 mg; I, 0.30 mg; Co, 0.14 mg; and Se, 0.30 mg.

^3^Calculated values unless indicated otherwise.

^4^CP=Crude protein.

^5^ME= Metabolizable energy.

**TABLE S2. Primer information of reference and target genes.**

| Gene symbol | Primer sequence(5'-3') | Amplicon length(bp) | Tm(°C) | Source |
| --- | --- | --- | --- | --- |
| *GPR41/FFAR3* | F: GCCCTTGCCCTTCATCTTCT | 136 | 60 | JQ776642.1 |
|  | R: CCGGGTCTTGTACCAGAGTG |  |  |  |
| *GPR43/FFAR2* | F: CAGAGGCAAAGAGACCGAGG | 83 | 60 | NM_001278758.1 |
|  | R: TGGTGAAGTCAGAACTCGGC |  |  |  |
| *GLP-2R* | F:GCCTCCATACTTACCTTGTGTGAG | 133 | 60 | NM 004246.1 |
|  | R:TTCTCGGAGCATCGGAGTCATC |  |  |  |
| *EGF* | F:TCTGAACCCGGACGGATTTG | 202 | 60 | NM_214020 |
|  | R:GACATCGCTCGCGAACGTAG |  |  |  |
| *EGF-R* | F:GGGATAGGGATTGGCGAGTT | 110 | 56 | Wang et al.2016[1] |
|  | R:TGCTACCGGCAGGATATGAAG |  |  |  |
| *TNF-α* | F: CCACGCTCTTCTGCCTACTGC | 168 | 60 | Feng et al.2015[2] |
|  | R: GCTGTCCCTCGGCTTTGAC |  |  |  |
| *IL-1β* | F:AGTGGAGAAGCCGATGAAGA | 113 | 60 | NM_214055.1 |
|  | R:CATTGCACGTTTCAAGGATG |  |  |  |
| *IL-8* | F:TAGGACCAGAGCCAGGAAGA | 92 | 60 | NM_213867.1 |
|  | R:AGCAGGAAAACTGCCAAGAA |  |  |  |
| *IFN-γ* | F: CCATTCAAAGGAGCATGGAT | 248 | 60 | [S63967](http://www.ncbi.nlm.nih.gov/entrez/query.fcgi?cmd=search&db=nucleotide&doptcmdl=genbank&term=S63967) |
|  | R: TTTTGATGCTCTCTGGCCTT |  |  |  |
| *AIP* | F:CTAAAGGGGCAGATGAATGG | 105 | 56 | Lackeyram et al.2010[3] |
|  | R:CACCTGTCTGTCCACGTTGT |  |  |  |
| *CK* | F:GAGCAAGCATCCCAAGTTCG | 169 | 60 | Wang et al.(2016)[1] |
|  | R:CTTCACACCATCCACCACCAG |  |  |  |
| *LDH* | F:AGCAAGAGGGAGAAAGCCGT | 148 | 56 | Wang et al.(2016)[1] |
|  | R:TTCCAAGCCACATAGGTCAAGA |  |  |  |
| *Bax* | F: AAGCGCATTGGAGATGAACT | 121 | 60 | Hong Chen et al.(2013)[4] |
|  | R: AAGCGCATTGGAGATGAACT |  |  |  |
| *Bcl2* | F: TGCCTTTGTGGAGCTGTATG | 144 | 60 | Hong Chen et al.(2013)[4] |
|  | R: GCCCGTGGACTTCACTTATG |  |  |  |
| *PPIA* | F: GGGAGAAAGGATTTGGTTAT | 175 | 62 | NM_214353 |
|  | R: ATGGACAAGATGCCAGGAC |  |  |  |
| *YWHAZ* | F:ATGCAACCAACACATCCTATC | 178 | 62 | Erkens et al. (2006)[5] |
|  | R: GCATTATTAGCGTGCTGTCTT |  |  |  |
| *B2M* | F:CAAGATAGTTAAGTGGGATCG | 161 | 60 | Wang et al.(2016)[1] |
|  | R:TGGTAACATCAATACGATTTC |  |  |  |
| *B-actin* | F:GGATGCAGAAGGAGATCACG | 134 | 51 | Wang et al.(2016)[1] |
|  | R:ATCTGCTGGAAGGTGGACAG |  |  |  |
| *HMBS* | F:AGGATGGGCAACTCTACCTG | 83 | 60 | Wang et al.(2016)[1] |
|  | R:GATGGTGGCCTGCATAGTCT |  |  |  |

**TABLE S3. Sequencing results of PCR products from the amplification of primers of reference and target genes designed for this experiment.**

| Genes | Sequences |
| --- | --- |
| *GPR41/FFAR3* | *TGGTACAAGACCCGGCCGAGGCCAGGACAGGCTGGACTGGTCAGCGTGGTCTG*  *CTGGCTCCTGGCTGCCGCTCACTGCAGTGTGGTCTACATCATCGAGTTCTCGGG* |
| *GPR43/FFAR2* | *GCCCTCATGGGTTTCGGCTACTACAGCAGCATCTACTGCAGCACGTGGCTCCTG*  *GCGGGCATCAGCATCGAGCGCTACCTGGGCGTGGCTTTCCCCGTGCAGAACAA*  *GCTGTCCCGCCGGCCCTTGT ACGGA* |
| *GLP-2R* | *CTTCTTCATCTTCCAGAAAATTCTCAAGCTTCACATTTCAAAGCTCAAAGCTCATC*  *AGATGTGCTTCAGAGATTATGAAT* |
| *EGF* | *GTTCATCCATTGGCAAAACCAGGAGCAAATCCCTGCTTACACCAAAATGGAGGCT*  *GTGAACATATCTGCAAAGAGAGTTTTGGAACTGCTC* |
| *EGF-R* | *TGCAAGTGGCAGAGAGCACATGGTCAGTTTTCCCTCGCGGTTGTTGGCCTGGAC*  *ATAGCAGTC* |
| *TNF-α* | *AAACTTTAAACAACAACAGCAACACTTAGAAAACAGGGATTCAGGAATGTGTGGCC*  *TGGACAACCAGGCACTGACCA* |
| *IL-1β* | *TTCACTATTGTCAAATCGATGCCCAGCTGTCTTCCCTAGATTAGTGCTAAGGAGT* |
| *IL-8* | *GAAATACGCATTCCACACCTTTCCACCCCAAATTTATCAAGGAACTGAGAGTGAT* |
| *IFN-γ* | *ATGTGATCAAGCAAGACATGTTTCAGAGATTCCTAAATGGTAGCGCTGGGAAACT*  *GAATGACTTCGAAAAGCTGATTAAAATTCCGGTTGATAATCTGCAG* |
| *AIP* | *GAAAAGCAGAAGCCCTGGCATGGACCGATTCCCGTACCTGGCTCTGTCCAAGA*  *CATACAACGTGGACAGACAGGTGA* |
| *CK* | *CGACGACATGATCCCCGCCCAGAAGTAGGAACCCAGCCGACTGCTGGAGCCC*  *AGGGCAAAGGTAGGACCAAGCCC* |
| *LDH* | *GCAGAGGAACGTCAATTTCTTCAAGTTCATCATCCCACAGATTGTCAAGTACAGT*  *CCTGACTGCATCATAATTG TGGTTTCGAACCCAGTGGATATTCTCACCTATGTTA*  *CCTGGAAGCTAAGTGGGTTACC* |
| *Bax* | *ATTTCCGAGTGGCGGCCGAAATGTTTGCTAACGGCAACTTCAACTGGGGCCGG*  *GTGGTCGCGCTGTTCTA* |
| *Bcl-2* | *GAGCGGCTCTTTGATTCTCCTGGCTGTCTCTGAGGCGCTGCTCAGTCTGGCCC*  *TGGTGGGAGCTTGCATCACCCTGGGTGCCTATCTGGGCCATAAGTGAAGTCCA*  *CGGGC* |
| *PPIA* | *TGATGCTTAGATATTCCAGGATTTATGTGCCAGGGTGGTGACTTCACACGCCATA*  *ATGGCACTGGTGGCAAGTCCATCTATGGAGAGAAATTTGATGATGAGAATTTTAT*  *CCTGAAGCATACGGGTCCTGGCATCTTGTCCAT* |
| *YWHA* | *GGGGGGCTGACTTACTTCTCTGTGTTCTATTATGAGATTCTGAACTCCCCAGAGA*  *AAGCCTGCTCTCTTGCAAAGACAGCATTTGATGAAGCCATTGCTGAACTTGATAC*  *ATTAAGTGAAGAGTCATACAAAGACAGCACGCTAATAATGCA* |
| *B2M* | *GCGATCGGGCTCCAGAGATTATGAGATGCTGCATCTGGGTTGGATGAATCCAAAT*  *TCTGATTTGTTGCTTTTTAATACTGATAAGCTTTTATACTTTATGCACATAAATCAGA*  *AATCGTATTGATGTTACCAAAC* |
| *B-actin* | *GGGGTGCGAGATGAGATCAGATCATCGCGCCTCCAGAGCGCAAGTACTCCGTGT*  *GGATCGGCGGCTCCATCCTGGCCTCGCTGTCCACCTTCCAGCAGATA* |
| *HMBS* | *GCGGAACTGAATTGAATGGTGCAGAGAGCATGCAAGAGACTATGCAGCGCCACT*  *CATCAG* |

**TABLE S4. Sequencing results of genes using BLASTN from NCBI against nucleotide collection.**

| Gene Name | Best hit in NCBI | Identity |
| --- | --- | --- |
| *PPIA* | Sus scrofa peptidylprolyl isomerase A (cyclophilin A) (PPIA), transcript variant X2, mRNA | 99% |
| *YWHAZ* | Sus scrofa 3-monooxygenase/tryptophan 5-monooxygenase activation protein, zeta (YWHAZ), transcript variant X1, mRNA | 100% |
| *B2M* | Sus scrofa beta-2-microglobulin (B2M), mRNA | 99% |
| *B-actin* | Sus scrofa actin, beta (ACTB), mRNA | 98% |
| *HMBS* | Sus scrofa hydroxymethylbilane synthase (HMBS), mRNA | 96% |
| *AIP* | Sus scrofa alkaline phosphatase, intestinal (ALPI), transcript variant 2, mRNA | 99% |
| *GPR41/FFAR3* | [Sus scrofa free fatty acid receptor 3 (FFAR3), mRNA](https://blast.ncbi.nlm.nih.gov/Blast.cgi#alnHdr_937576150) | 98% |
| *GPR43/FFAR2* | [Sus scrofa free fatty acid receptor 2 (FFAR2), mRNA](https://blast.ncbi.nlm.nih.gov/Blast.cgi#alnHdr_523580027) | 98% |
| *GLP-2R* | [Sus scrofa glucagon like peptide 2 receptor (GLP2R), mRNA](https://blast.ncbi.nlm.nih.gov/Blast.cgi#alnHdr_350536456) | 96% |
| *EGF* | [Sus scrofa epidermal growth factor (EGF), mRNA](https://blast.ncbi.nlm.nih.gov/Blast.cgi#alnHdr_47522861) | 99% |
| *EGF-R* | Sus scrofa epidermal growth factor receptor (EGFR), mRNA | 98% |
| *TNF-α* | [Sus scrofa tumor necrosis factor (TNF), mRNA](https://blast.ncbi.nlm.nih.gov/Blast.cgi#alnHdr_47522865) | 97% |
| *IL-1β* | [Sus scrofa interleukin 1, beta 2 (IL1B2), mRNA](https://blast.ncbi.nlm.nih.gov/Blast.cgi#alnHdr_1331383532) | 98% |
| *IL-8* | [Sus scrofa interleukin-8](https://blast.ncbi.nlm.nih.gov/Blast.cgi#alnHdr_516196), mRNA | 100% |
| *IFN-γ* | [Sus scrofa interferon gamma mRNA, complete cds](https://blast.ncbi.nlm.nih.gov/Blast.cgi#alnHdr_289655664) | 100% |
| *AIP* | Sus scrofa intestinal-type alkaline phosphatase-like (LOC100521229), mRNA | 98% |
| *CK* | Sus scrofa creatine kinase, M-type (CKM), transcript variant X1, mRNA | 96% |
| *LDH* | Sus scrofa lactate dehydrogenase B (LDHB), transcript variant X2, mRNA | 97% |
| *Bax* | Sus scrofa BCL2 associated X, apoptosis regulator (BAX), transcript variant X2, mRNA | 97% |
| *Bcl-2* | Sus scrofa BCL2, apoptosis regulator (BCL2), transcript variant X1, mRNA | 97% |

**TABLE S5. Sequences from the samples of all groups^1^.**

| Sample ID | Valid Sequences | 3﹪distance | | | | |
| --- | --- | --- | --- | --- | --- | --- |
|  |  | Average-Length(bp) | Core OTUs | Shannon | Simpson | Coverage (%) |
| 0-A | 5080 | 1,491.20 | 33 | 4,35 | 0,065 | 92,90 |
| 0-B | 4875 | 1,489.10 | 27 | 4,24 | 0,074 | 90,07 |
| 0-C | 4100 | 1,494.20 | 31 | 4,72 | 0,051 | 90,17 |
| 0-D | 4160 | 1,485.90 | 26 | 4,18 | 0,066 | 89,81 |
| 0-E | 4951 | 1,486.00 | 28 | 4,02 | 0,062 | 92,58 |
| 7-A | 4080 | 1,493.80 | 45 | 5,62 | 0,029 | 89,66 |
| 7-B | 4032 | 1,484.50 | 47 | 4,98 | 0,021 | 90,90 |
| 7-C | 4421 | 1,492.00 | 51 | 5,22 | 0,028 | 91,04 |
| 7-D | 4900 | 1,487.20 | 38 | 4,92 | 0,025 | 90,74 |
| 7-E | 5100 | 1,477.60 | 50 | 5,58 | 0,026 | 93,75 |
| 14-A | 5620 | 1,480.90 | 37 | 4,13 | 0,029 | 95,49 |
| 14-B | 5127 | 1,493.30 | 29 | 4,85 | 0,042 | 91,81 |
| 14-C | 5018 | 1,479.80 | 41 | 4,76 | 0,037 | 90,43 |
| 14-D | 4225 | 1,482.50 | 42 | 5,22 | 0,039 | 90,30 |
| 14-E | 6373 | 1,487.50 | 38 | 4,80 | 0,031 | 91,75 |
| 21-A | 4091 | 1,481.40 | 28 | 5,09 | 0,047 | 89,25 |
| 21-B | 5050 | 1,485.50 | 42 | 4,41 | 0,039 | 90,31 |
| 21-C | 5072 | 1,490.60 | 36 | 4,38 | 0,035 | 95,53 |
| 21-D | 5034 | 1,488.50 | 43 | 4,86 | 0,037 | 93,73 |
| 21-E | 6130 | 1,485.40 | 33 | 4,74 | 0,036 | 96,15 |

**TABLE S6.** Relative abundances of the top five predominant phyla, classes and families of jejuna microbiota of weaned-piglets.

| **Growth stages** | **(Mean relative abundance, %, n=5)** | | |
| --- | --- | --- | --- |
|  | Dominant Phyla | Dominant Classes | Dominant families |
| **PW day 0** | *Firmicutes(64.57)* | *Clostridia(43.05)* | *Ruminococcaceae(14.31)* |
|  | *Tenericutes(15.58)* | *Bacilli(15.58)* | *Lactobacillaceae(13.52)* |
|  | *Bacteroidetes(11.67)* | *Bacteroidia(11.66)* | *Clostridiaceae(11.98)* |
|  | *Proteobacteria(6.62)* | *Negativicutes(7.96)* | *Prevotellaceae(6.69)* |
|  | *Actinobacteria(0.25)* | *Mollicutes (7.20)* | *Lachnospiraceae(6.52)* |
| **PW day 7** | *Firmicutes(84.74)* | *Clostridia(40.16)* | *Lachnospiraceae(17.09)* |
|  | *Bacteroidetes(11.07)* | *Bacilli(34.22)* | *Ruminococcaceae(8.79)* |
|  | *Tenericutes(2.46)* | *Bacteroidia(11.07)* | *RF9_norank(8.58)* |
|  | *Proteobacteria(0.82)* | *Negativicutes(8.38)* | *S24-7(8.04)* |
|  | *Actinobacteria(0.45)* | *Erysipelotrichia(7.16)* | *Erysipelotrichaceae(7.16)* |
| **PW day 14** | *Firmicutes(84.45)* | *Clostridia(62.33)* | *Ruminococcaceae(27.56)* |
|  | *Bacteroidetes(6.13)* | *Bacilli(14.44)* | *Lactobacillaceae(12.13)* |
|  | *Tenericutes(5.94)* | *Bacteroidia(6.11)* | *RF9_norank(10.54)* |
|  | *Proteobacteria(1.62)* | *Erysipelotrichia(5.94)* | *Lachnospiraceae(9.60)* |
|  | *Actinobacteria(0.45)* | *Negativicutes(5.09)* | *Clostridiaceae(7.85)* |
| **PW day 21** | *Firmicutes(87.46)* | *Clostridia(59.15)* | *Ruminococcaceae(25.89)* |
|  | *Bacteroidetes (8.04)* | *Bacilli(19.97)* | *Clostridiaceae(12.59)* |
|  | *Tenericutes(2.04)* | *Bacteroidia(8.84)* | *Lactobacillaceae(11.66)* |
|  | *Proteobacteria(1.86)* | *Negativicutes(2.68)* | *Lachnospiraceae(9.80)* |
|  | *Actinobacteria(0.42)* | *Erysipelotrichia(4.86)* | *Erysipelotrichaceae(4.86)* |

**TABLE S7.** The family-level taxonomy of abundant core OTUs of jejunal microbiotas in weaned-piglets (n=5).

| **Post-weaning age** | **Genera of the core OTUs* of jejunal microbiotas (% of piglets with OTUs)** |
| --- | --- |
|  | Core microbiota |
| **PW day 0** | *Ruminococcaceae (100), Lactobacillaceae (100), Lachnospiraceae (100), Clostridiaceae (100), Prevotellaceae (80), Clostridiales_xiii_incertae_sedis (80), Veillonellaceae (80).* |
| **PW day 7** | *Ruminococcaceae (100), Lactobacillaceae (100), Lachnospiraceae (100), Clostridiaceae (100), RF9_norank (100), S24-7 (80), Acidaminococcaceae (80), Erysipelotrichaceae (100), Streptococcaceae (80).* |
| **PW day 14** | *Ruminococcaceae (100), Lactobacillaceae (100), Lachnospiraceae (100), Clostridiaceae (100), RF9_norank (80), Acidaminococcaceae (80), Erysipelotrichaceae (100).* |
| **PW day 21** | *Ruminococcaceae (100), Lactobacillaceae (100), Lachnospiraceae (100), Clostridiaceae (100), RF9_norank (80), Acidaminococcaceae (80), Erysipelotrichaceae (100), Streptococcaceae (80), Prevotellaceae (100).* |

**Note:** * Present at >2 % relative abundance, PW=post weaning.

**References**

1. Wang S, Guo C, Zhou L, Zhong Z, Zhu W, Huang Y, et al. Effects of dietary supplementation with epidermal growth factor-expressing Saccharomyces cerevisiae on duodenal development in weaned piglets. *Br J Nutr*. 2016; 115(9):1509-1520.

2. Feng Z, Li T, Wu C, Tao L, Blachier F, Yin Y. Monosodium L-glutamate and dietary fat exert opposite effects on the proximal and distal intestinal health in growing pigs. *Appl Physiol Nutr Metab.* 2015; 40(4):353-363.

3. Lackeyram D, Yang C, Archbold T, Swanson KC, Fan MZ. Early weaning reduces small intestinal alkaline phosphatase expression in pigs. *J Nutr*. 2010; 140(3):461-468.

4. Chen H, Mao X, He J, Yu B, Huang Z, Yu J, et al. Dietary fibre affects intestinal mucosal barrier function and regulates intestinal bacteria in weaning piglets. *Br J Nutr*. 2013; 110(10):1837-1848.

5. Erkens T, Van Poucke M, Vandesompele J, Goossens K, Van Zeveren A, Peelman LJ, et al. Development of a new set of reference genes for normalization of real-time RT-PCR data of porcine backfat and longissimus dorsi muscle, and evaluation with PPARGC1A. *BMC Biotechnol.* 2006; 6(1):41.
