# Supplementary material for: Microbial-Driven Butyrate Regulates Jejunal Homeostasis in Piglets During the Weaning Stage
Source: Front Microbiol. 2019 Jan 18;9:3335. doi: 10.3389/fmicb.2018.03335 (PMC6345722; doi:10.3389/fmicb.2018.03335)
Supplement: Supplementary file 2 [file Data_Sheet_2.docx]

**Supplementary file 2**


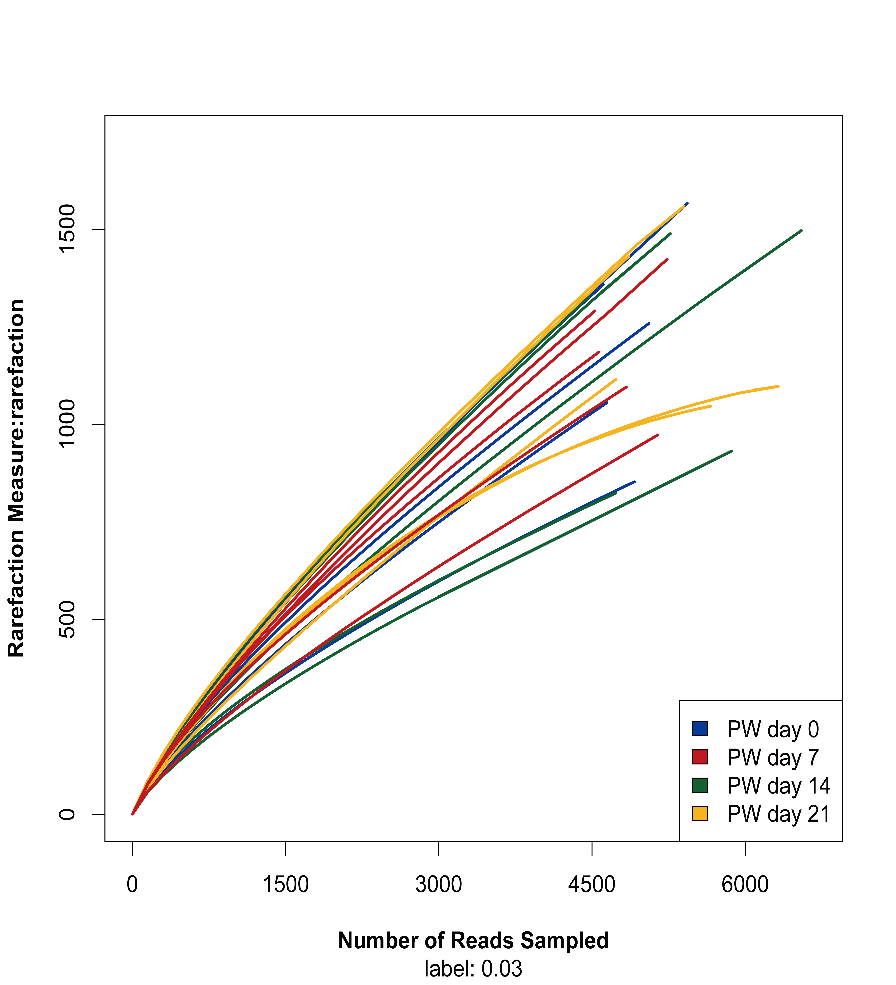


**FIGURE S1. Rarefaction curves for all twenty samples of the jejunum in weaned piglets.**

**Note:** PW: post-weaning.

**
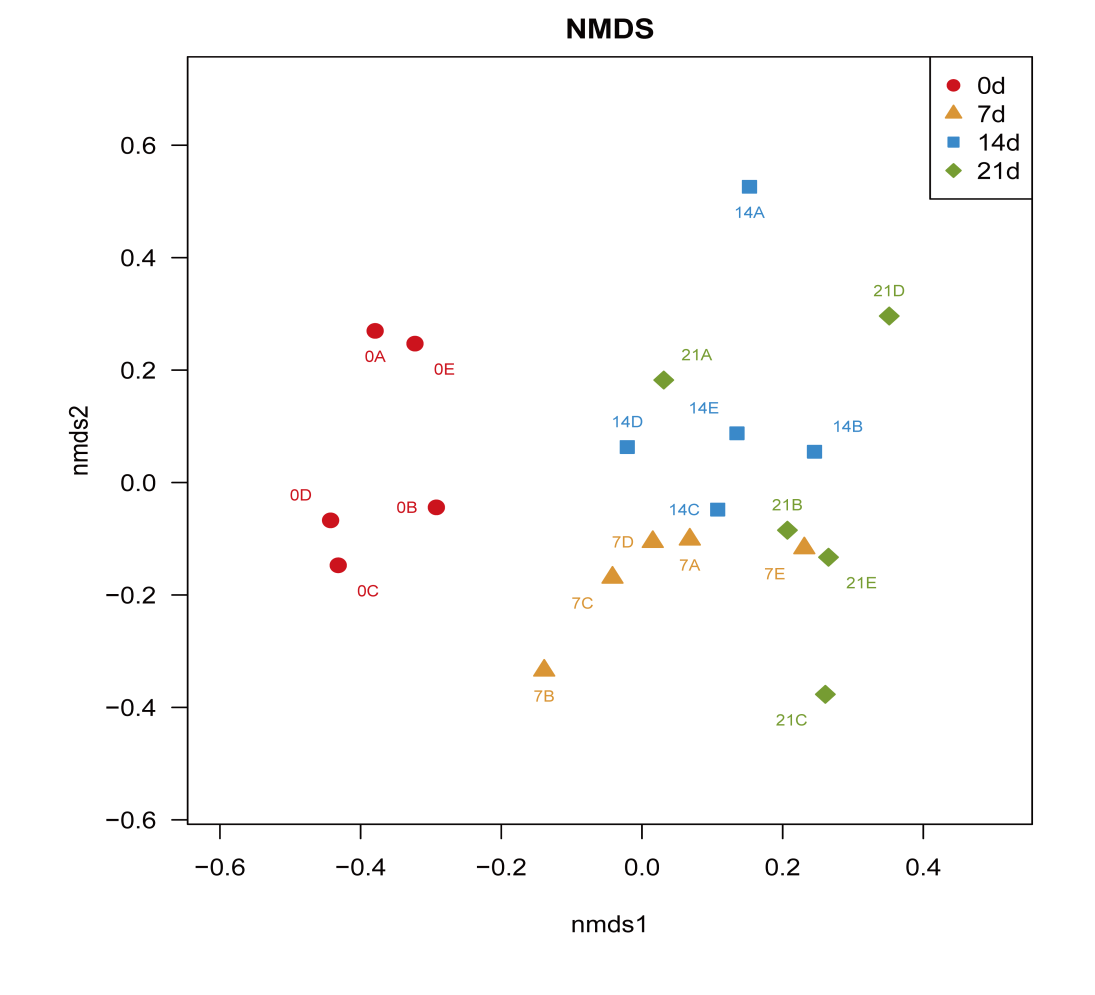
**

**FIGURE S2. Non-metric multidimensional scaling (NMDS) plots for microbial distributions with different size fractionations based on similarity or dissimilarity at post-weaning the three weeks.**

**Note:** 0d: post-weaning day 0, 7d: post-weaning day 7, 14d: post-weaning day 14, 21d: post-weaning day 21.


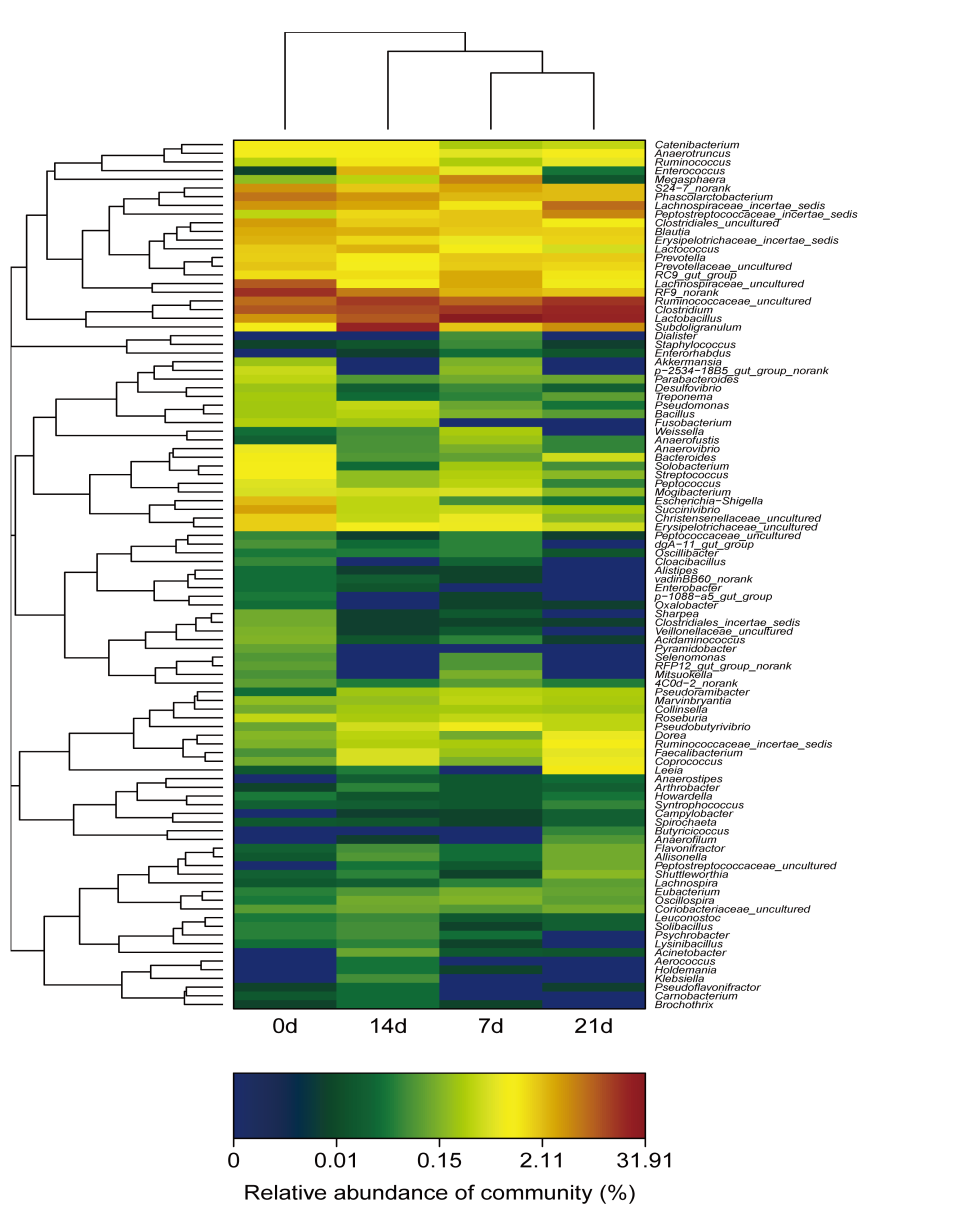


**FIGURE S3. Bacterial distribution of the heatmap at genus level (n=5).**

**Note:** Double dendrogram showed the bacterial distribution (genus level) among all samples. The bacterial phylogenetic tree was calculated using the neighbor-joining method, and the relationship among samples was determined using bray distance and the complete clustering method. The relative abundance higher than 0.1% within total bacteria was sorted for the analysis. The heatmap plot depicts the relative percentage of each bacteria (variables clustering on the Y-axis) within each sample (X-axis clustering). The relative values for the bacteria are depicted by color intensity in the legend indicated at the top of the figure. Clusters based on the distance of the different samples along the X-axis and bacteria along the Y-axis are indicated at the top and bottom of the figure, respectively.


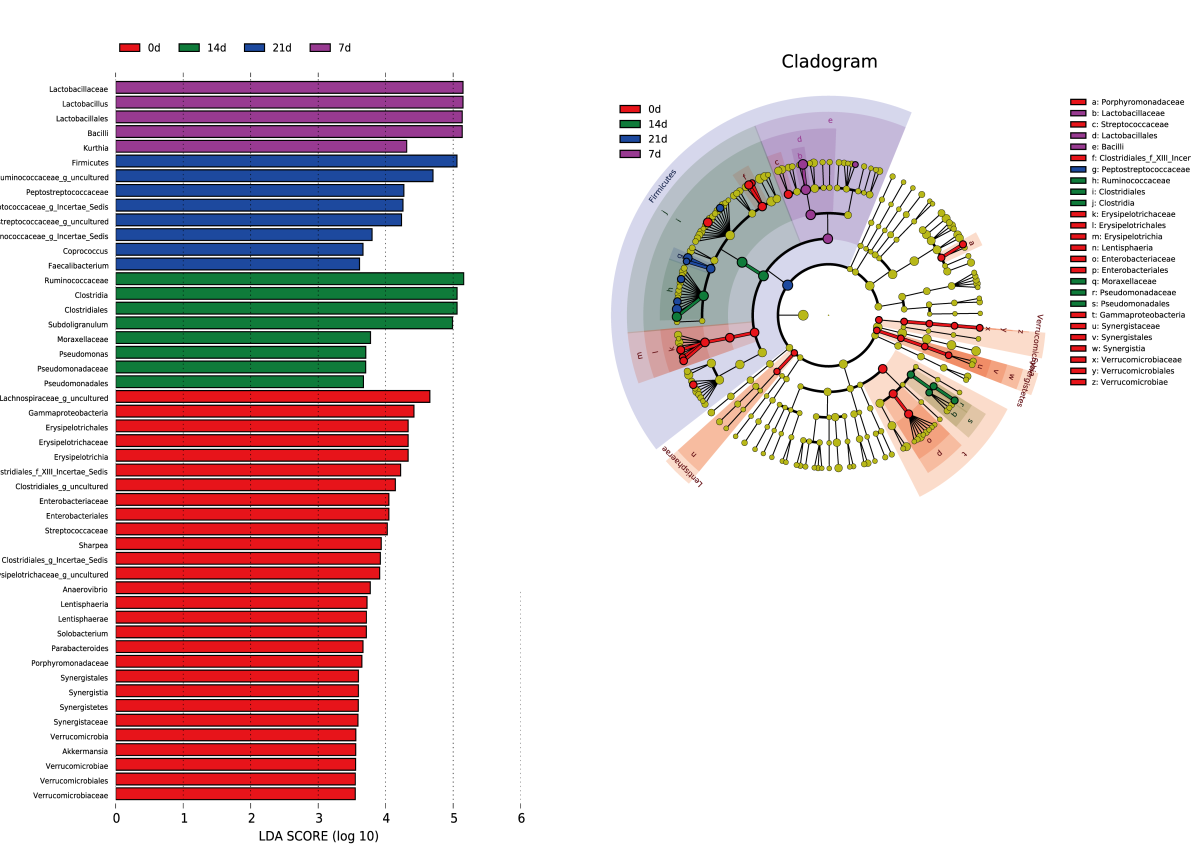


**FIGURE S4. Cladogram of bacterial biomarkers associated with phase of production (LEfSe) (n=5).**

Note: Samples were grouped into post-weaning day 0, 7, 14 and 21, respectively. This hierarchal tree of taxonomical nodes, where diameter of the nodes indicates relative abundance, showed jejunal biomarkers for the post-weaning the first 3 weeks of life. Differences are represented by the color of the most abundant class. The diameter of each circle’s diameter is proportional to the taxon’s abundance.
